# Safety and Efficacy of Medicinal Plants Used to Manufacture Herbal Products with Regulatory Approval in Uganda: A Cross-Sectional Study

**DOI:** 10.1155/2022/1304839

**Published:** 2022-04-13

**Authors:** Bruhan Kaggwa, Henry Kyeyune, Edson Ireeta Munanura, Godwin Anywar, Stephen Lutoti, Jacqueline Aber, Lynn K. Bagoloire, Anke Weisheit, Casim Umba Tolo, Pakoyo Fadhiru Kamba, Patrick Engeu Ogwang

**Affiliations:** ^1^Mbarara University of Science and Technology, Pharm-Bio Technology and Traditional Medicine Center (PHARMBIOTRAC), P.O. Box 1410, Mbarara, Uganda; ^2^Makerere University, College of Health Sciences, Department of Pharmacy, P.O. Box 7062, Kampala, Uganda; ^3^Makerere University, Department of Plant Sciences, Microbiology and Biotechnology, P.O. Box 7062, Kampala, Uganda; ^4^Makerere University, College of Health Sciences, School of Medicine, Clinical Epidemiology Unit, P.O. Box 7072, Kampala, Uganda

## Abstract

**Introduction:**

The Uganda National Drug Authority requires phytochemical screening, freedom from microbial contamination, and evidence of safety and efficacy of the constituent plants to register herbal products. Since Uganda has no pharmacopeia, safety, efficacy, and plant processing information are not readily available. We documented the plant materials used to manufacture products in Uganda and established evidence of their safety and efficacy and availability of monographs.

**Methods:**

The NDA register of herbal products was reviewed, and a product list was extracted. The herbal products were purchased from local pharmacies, and their labels were studied to identify plant ingredients and drug use. Literature was reviewed to document evidence of the safety and efficacy of the plant materials concerning manufacturer's claims. Also, the WHO and available African Pharmacopeia were searched to establish the availability of the plant monographs.

**Results:**

Of the 84 NDA-registered local products, only 18 were obtained from the market; 82% were indicated for respiratory tract disorders. Thirty-three plant materials were listed with *Eucalyptus globulus* Labill, being the commonest. Several *in vitro* and *in vivo* studies demonstrate efficacy, thus supporting the use of the selected plant species for empirical treatment as stated on the product label. While most plants were safe, some species such as *Albizia coriaria* Oliv. had dose-dependent toxicities that cannot be predicted in combinations. The WHO, African Pharmacopoeia, and West African Herbal Pharmacopoeia had only 16 plant monographs of the 33 plants of interest. Nevertheless, *Aloe vera* (L.) Burm.f., *Azadirachta indica* A.Juss., *Zingiber officinale* Roscoe, and *Allium sativum* L. monographs were published by all three pharmacopoeias.

**Conclusions:**

Preclinical evidence of safety and efficacy exists in the literature for most of the plants used to manufacture registered herbal products in Uganda. More specific bioassays and clinical trials are required for the products to provide conclusive evidence of safety and toxicity. Monographs are urgently needed for the Ugandan plants.

## 1. Introduction

Traditional herbal medicines have been defined as naturally occurring, plant-derived substances with minimal or no industrial processing. Such substances have been used to treat illness within local or regional healing practices [1]. More than 80% of the population within developing countries relies on herbal and other traditional medicines for primary health care [2]. These medicines are readily available, affordable, and perceived to be safe.

Traditionally, medicinal plant products have been prescribed by traditional healers within communities or self-prescribed. In this regard, assessment of efficacy and safety was based on the history of use and informal knowledge passed on from generation to generation. However, with commercialization, there is a need for scientific proof that the products are effective and safe. Various studies have demonstrated deficiencies in the quality of herbal medicines; from adulteration with conventional medicines to substitution with ineffective or harmful plant materials and contamination with soil, microorganisms, pesticides, aflatoxins, etc. [3].

Consequently, the developed world's regulatory authorities have established policies to regulate herbal and other traditional medicines. The US FDA and the EMA, for instance, have published strict requirements for market authorization of traditional methods; as such, the manufacturer has to demonstrate the efficacy and safety of the product through clinical trials, a quality assurance plan for cultivation and harvesting of the plant materials as well as protocols for good manufacturing practices [4, 5].

In contrast, traditional medicines are hardly regulated in developing countries. In a study to evaluate the regulatory framework of traditional medicines, over 60% of developing countries did not have any framework in place [6]. Following this study, the WHO published several guidelines for quality assurance of herbal medicines including, but not limited to, guidelines for registration of traditional medicines in the African region [7], guidelines for assessing the quality of herbal medicines [8], and the guidelines for good manufacturing practices herbal medicines [9].

Many African countries still have weak regulatory frameworks for traditional medicines despite these guidelines. In Uganda, for instance, there are no herbal pharmacopoeial methods for assessing the quality of herbal medicinal products. Consequently, all the locally manufactured herbal products authorized for marketing are not fully registered but rather “notified” [10]. This is because the medicines have not been evaluated fully for quality, safety, and efficacy due to regulatory and manufacturer inadequacies. To notify herbal products, the NDA considers long-standing folk use as evidence for the safety of any herbal plant material and therefore waives thorough toxicological evaluation of the product concerning the plant species used. In addition, there are no specific requirements regarding the product's efficacy. Only a report summarizing results of phytochemical screening of the plant materials used is required [11]. Once evidence for traditional use is established, specific preclinical and clinical studies to establish the safety and efficacy of the herbal products used are not a prerequisite to obtain market authorization.

Unlike in Western medicines in which pure bioactive compounds are used in pharmaceutical products, traditional medicine utilizes crude forms of whole plants or plant parts. The various plants and herbal products utilized in traditional/herbal medicine differ due to differences in climate and culture [12, 13]. Consequently, analytical methods for herbal products developed in one country are not applicable in another country. Therefore, monographs developed by countries with established herbal medicine regulatory systems such as China and the USA cannot be applied to products that are local to a culturally and climatically different region such as sub-Saharan Africa [14].

This study aimed to document the plant raw materials used in Uganda for the manufacture of herbal products and establish a database of safety and efficacy information. This information can be added to future herbal pharmacopeia/monographs for routine use in quality assurance and quality assessment by manufacturers and regulators, respectively.

## 2. Methods

### 2.1. Study Design and Setting

This was a cross-sectional study of local herbal products registered and sold in Uganda in 2019 and a review of published literature on the safety and efficacy of the herbal raw materials between 1960 and 2021. The products were bought from the busy cities of Mbarara, Jinja, Wakiso, and Kampala. In addition, most of the manufacturers are located in this area. The pharmacies were accessed conveniently, and the only important factor was the presence of the products of interest.

### 2.2. Data Collection Methods

Data were collected mainly by desk review. Drug registers, product labels, and journal articles were considered. The data were collected as mentioned in the following sections.

#### 2.2.1. Identintification of the Most Common Raw Materials and Indicated Use of the Products

The most current drug register (August 2019) of herbal medicinal products was obtained from the NDA website (https://www.nda.or.ug/drug-register-downloads/). Then, a list of the notified (partial registration to permit marketing) local herbal medicinal products was compiled. A sample of each of the products on the notified list was purchased from local pharmacies.

After the products were purchased, their labels were studied to identify the indications (use of the drug) and the active ingredients (medicinal plant materials). A database of all the herbal plant materials was then developed, and the materials were ranked according to their popularity (frequency of appearance) in the herbal medicinal products.

#### 2.2.2. Establishment of Evidence for Efficacy and Safety of Plant Materials and Rationale for the Polyherbals

We conducted a literature review to obtain evidence of biological activity concerning the current traditional use (according to the manufacturers' labels) and safety of all the plant species used. We searched databases and search engines such as PubMed, Google Scholar, ResearchGate, Web of Science, and Scopus. The search terms consisted of the scientific plant names, pharmacological/therapeutic activity of interest, and toxicity. Only full-length articles published in English were reviewed.

#### 2.2.3. Search for Plant Monographs Already Available

Since there are no Ugandan monographs or pharmacopeia, we were interested in finding out if monographs of the plants used in Uganda have been published in other African countries. The uses and composition of the plants were considered to be similar due to their similarity in culture and climate. We reviewed the African Pharmacopoeia 2014 [15] and the West African Pharmacopoeia 2013 [16]. Due to the absence of the monographs for most of the plants in the African Pharmacopeia, we also searched WHO monographs on selected medicinal plants volumes I–IV published between 1999 and 2009 [17–20].

## 3. Results and Discussions

### 3.1. Identification of the Most Common Plant Raw Materials and Indicated Use of the Products

By 31^st^ March 2019, the NDA drug register had 4,148 and 214 conventional products and herbal medicinal products, respectively. All the herbal products are not fully registered but rather notified, and the majority (130) was imported, mainly from India and China. 84 locally manufactured herbal products were notified. Of these, 82% (*n* = 69) were syrups or solutions indicated for systemic treatment of upper respiratory tract disorders, such as cough, asthma, and flu. For skin infections and wound healing, the others were ointments, creams, balms, or soaps. Three products were indicated for the treatment of peptic ulcers (Table 1).

Although there were 84 locally manufactured products, we could only find 18 products in the study areas. We evaluated these products to obtain the most commonly used medicinal plant species in the formulations. Two products that did not indicate the active ingredients were eliminated.

A review of the products revealed that 33 plant species were used as raw materials. Whereas various scientific names of the plant species and/or their synonyms were used on the product information labels, we adopted the approved scientific names and their authorities according to the Kew database; Plants of the World http://www.plantsoftheworldonline.org accessed on 19^th^ March 2021. This ensured uniformity and maintained scientific standards. We did not verify from the manufacturers if the plants indicated on the labels were the ones used to manufacture the products.

**Figure 1:**
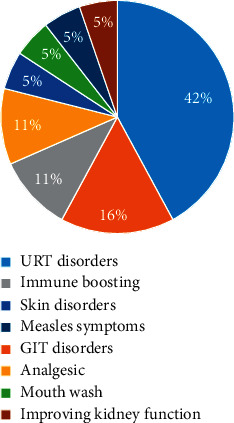
Summary of diseases treated by the herbal medicinal products manufactured in Uganda.

Table 1 summarizes the products found in pharmacies during data collection. Indications are as written on the product label (these are classified into major disease categories (Figure 1). Where wrongly spelled scientific plant names were found on the product labels, corrected and verified names were used instead.

**Figure 2:**
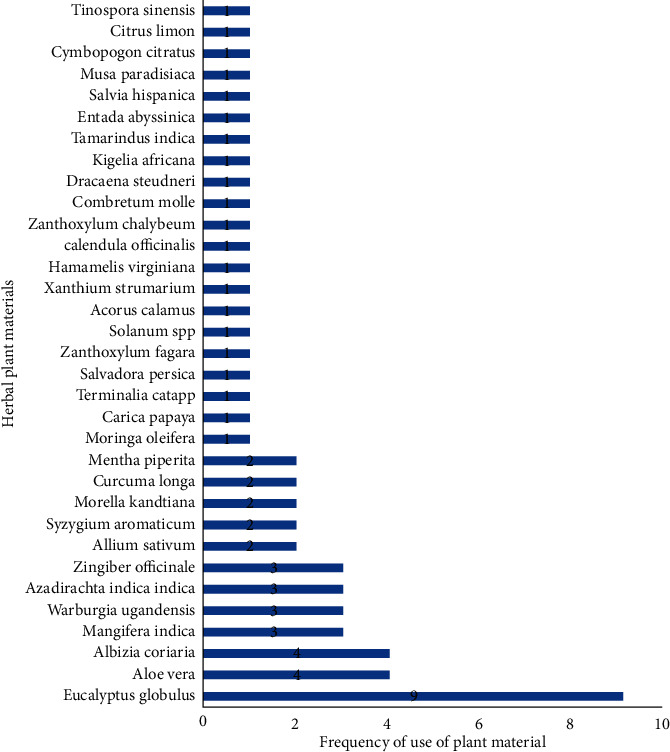
Popularity of herbal materials used to manufacture local herbal medical products in Uganda.

Of the 33 plants listed, *Eucalyptus globulus* was by far the most used material. *E. globulus* was mentioned in 50% (*f* = 9) of the products, followed by *Aloe barbadensis*, *Albizia coriaria*, *Mangifera indica,* and *Warburgia ugandensis* tied in the third place (Figure 2). Most of the plant materials are used for self-limiting conditions, majorly to alleviate the signs and symptoms of upper respiratory tract disorders (URT) and gastrointestinal tract (GIT) disorders (Figure 1).

Figure 1 summarizes the major diseases treated by locally manufactured herbal products. Over 40% of the medicines are used to treat upper respiratory tract disorders (whooping cough, catarrh, sore throat, asthma, bronchitis, cough, flu, measles symptoms, allergic cough, smokers cough, productive cough, lung cleaning, sinusitis, common colds, rhinitis, allergic conditions, bronchial congestion, painful coughing, dry/irritating cough, nasal congestion). The rest of the products are indicated for gastrointestinal disorders (cleanses GIT, gastric ulcers, stomach ulcers, flatulence, constipation, ulcers); immune boosting (boost the immune system, antioxidant); analgesic (pain relief, painful lips); skin disorders (relief of minor skin irritations due to; insect bites, minor cuts, and minor scrapes); and mouth wash (toothache, bad odor, sensitivity, bleeding gums, cavities, tooth decay). Most of these conditions are similar to those the folk/traditional uses indicated in the pharmacopeia/monographs reviewed (Table 2).

Figure 2 shows the popularity of the plant raw materials for manufacture of herbal products. The plants were ranked based on the number of times they appear on the product label as active ingredients. Of the 33 plant raw materials, *Eucalyptus globulus* was the most common, followed by *Aloe vera* and *Albizia coriaria* in the second place, while *Albizia coriaria*, *Mangifera indica*, *Warburgia ugandensis*, *Azadirachta indica*, and *Zingiber officinale* tied in the third place. The rest of the plants were only mentioned once.

### 3.2. Establishment of Evidence for Efficacy and Safety of Plant Materials and Rationale for the (Poly) Herbal Products

Half (*n* = 8) of the surveyed locally produced herbal products were for the management of diseases of the respiratory system, with label claims such as dry, wet, and whooping cough, sore throat, bronchial asthma, nasal congestion, flu, common cold, sinusitis, and allergies. Digestive system conditions such as gastric ulcers, flatulence, and constipation are also frequently targeted. Others include immune boosters, mouth wash, antioxidants, wounds, allergies, and a lip balm. Nine of the most frequently used plant materials were reviewed in detail, for evidence of safety and efficacy in managing the conditions on the label claim. These were *A. coriaria, M. indica, A. indica, S. aromaticum, A. barbadensis, W. ugandensis, Z. officinale, A. sativum*, and *E. globulus.* The information for the rest of the plant materials is summarized in Table 3.

To establish the safety and efficacy of the plant materials, we adopted the recommendations of the WHO [7] as mentioned in the following sections.

#### 3.2.1. General Requirements for Safety

In addition to authentication of the plant species, biological information on the safety of the plant/product should be provided. If biological evidence is lacking, data documented from long-term use should be used to assess the risk. However, the absence of toxicity data is not a guarantee of safety. Toxicity studies are required for plants and products thereof with a known toxicological risk.

#### 3.2.2. General Requirements for Efficacy

Generally, proof of the efficacy of traditional medicine is determined by indications for use (concerning biological evidence) and testimony by physicians, traditional practitioners, and patients after a long duration of use. Appropriate clinical trials should be conducted for cases where no history of use or biological information has been documented. The WHO has published guidelines for the clinical study of traditional medicines in the WHO African region [79].

To ensure reproducibility of efficacy, the preparation of medicines should be standardized so that each batch contains a defined amount of the active ingredients.

Information on safety and efficacy can be obtained from scientific literature, national or international pharmacopeia, and WHO monographs for selected plant materials.

#### 3.2.3. *Albizia coriaria* Oliv. (Fabaceae)

*Albizia coriaria* is used in many local herbal preparations to manage upper respiratory tract infection (URTI) and other infections in Uganda [21, 80]. Products containing *A. coriaria* include Gabogola syrup®, Kwesiima cough mixture®, Muwereza herbal cough remedy®, and Phycof cough syrup®. Upper respiratory tract infections are typically caused by the rhinovirus, adenovirus, influenza, enterovirus, and respiratory syncytial virus [81]. Bacteria cause about 15% URTI, and the most implicated bacterium is *Streptococcus pyogenes*. URTI causes a variety of diseases with overlapping presentations, including acute bronchitis, influenza, rhinitis, pharyngitis, tonsillitis, laryngitis, and other respiratory distress syndromes. Symptoms of URTI commonly include cough, sore throat, runny nose, nasal congestion, headache, low-grade fever, facial pressure, sneezing, malaise, and myalgias. Most viral infections are self-limiting [81]. The goal of treatment is symptom relief, and in case of bacterial infections, anti-infectives can be used [82]. Therefore, for the effective management of URTI, the medication should possess one or more of the following activities: antiviral, antibacterial, anti-inflammatory, expectorant, and decongestant.

Bioactive compounds such as lupeol, lupenone, betulinic acid, and catechin extracted using ethyl acetate from the stem bark of *A. coriaria* had activity against *Pseudomonas aeruginosa* and *Escherichia coli* [83]. These bacteria are, however, not commonly implicated in the causation of URTI. No specific studies were evaluating the activity of the bark of *A. coriaria* against Gram-positive cocci, which are the causative agents of URTI. Lupeol has anti-inflammatory activity [84]. Treatment with lupeol reduces mucus secretion and overall lung inflammation in a murine model [85]. The antiviral activity of lupenone is selective. Lupenone exhibits a strong viral plaque inhibitory effect against herpes simplex virus (HSV)-1 and HSV-2 and inhibits African swine fever virus [86], but none of these viruses cause URTI. When studied in experimentally induced polymicrobial sepsis in mice, betulinic acid exhibited a protective effect on the lungs by inhibiting the production of inflammatory mediators. In another study, betulinic acid showed anti-inflammatory and antioxidant properties that protect the lungs against lipopolysaccharide-induced lung inflammation in rats [87].

Herbalists use *A. coriaria* in very small quantities in polyherbal preparations and boil it for several hours because of its known toxicity. Not surprisingly, the DMSO and ethanol extracts of *A. coriaria* were highly cytotoxic to the human glioblastoma U87.CD4.CXCR4 cells (CC_50_ = 6.4 and <4 *μ*g/ml respectively) [57]. *Albizia* spp. has a toxic compound, 4-methoxypyridoxine, which antagonizes vitamin B_6_ [88].

Other *Albizia* species have shown different toxicities, including several central nervous system disturbances and other effects on the cardiovascular and respiratory systems. For instance, seed methanolic extracts of *Albizia greveana* and *Albizia bernieri* are toxic to mice with LD_50_ of 1.13–2.3 and 52 mg/kg body weight, respectively, the former being more than one toxic [89]. However, the methanol and aqueous extracts of *A. coriaria* have been shown to have very low cytotoxicity (CC_50_ >500 *μ*g/ml) against human embryonic lung fibroblast (HELF) cells [90]. In contrast, the methanol, ethanol, ethyl acetate, and diethyl ether extracts of *A. coriaria* were not toxic to the human keratinocyte cell line (HaCaT) IC_50_ > 512 [91].

There is scientific evidence for the usefulness of *A. coriaria* in the symptomatic management of respiratory tract conditions. However, specific studies need to be done to assess URTI activity against the causative organisms. Specific toxicity studies on the bark of *A. coriaria* need to be done to conclude on the safety of the plant.

#### 3.2.4. *Mangifera indica* L. (Anacardiaceae)

*Mangifera indica* is also used in many preparations for URTI management from the survey, including Gabogola syrup®, Kwesiima cough mixture®, and Yeco cough doctor®. *M. indica* is widely used to manage various infections and as an immune booster in Uganda [80]. Various pharmacological investigations have been carried out to explore the therapeutic potential of the phytochemicals in different plant parts of *M. indica* [92]. However, for the management of URTI, the antiviral, antibacterial, anti-inflammatory, expectorant, and decongestant activities of *M. indica* are important. Extracts from the fruit pulp of *M. indica* possess antiviral activity against influenza virus H9N2 and chicken embryo fibroblast (CEFs) with low cytotoxicity [93]. Mangiferin, extracted from the leaves of *M. indica*, inhibited herpes simplex virus 2 (HSV-2) replication but did not directly inactivate the virus [94]. Extracts from the stem bark of *M. indica* have some bacteriostatic activity against S. *aureus* albeit lower than the activity of the positive control used, ampicillin [95]. Alok et al. [96] demonstrated the antibacterial effects of the aqueous kernel extracts of two *M. indica* varieties (Bagnapalli and Senthura) from Tamil Nadu (India) against *Staphylococcus aureus* and *Pseudomonas aeruginosa.* Acetone, methanol, and water extracts of the leaves on *M. indica* exhibited lower antibacterial activity against *S. aureus, S. pyogenes, S. pneumoniae, Bacillus cereus, E. coli, P. aeruginosa, Proteus mirabilis, Salmonella typhi*, and *Shigella flexneri* using the agar well (cup plate) diffusion method than gentamicin and erythromycin [97].

A toxicological evaluation of *M. indica* leaf extract containing 60% mangiferin revealed a no-observed-adverse-effect level in male and female rats at 2000 mg/kg body weight/day. Additionally, no evidence of genotoxicity was found in a bacterial reverse mutation test [98]. *In vivo* acute toxicological studies of the stem bark aqueous extract of *M. indica* in rats and mice showed no lethality at the limit dose of 2,000 mg/kg body weight and no adverse effects when given by oral or dermal administration. Deaths occurred when the extract was administered intraperitoneally at 200 mg/kg in mice [99]. *M. indica* extracts were nonirritating on rabbits' skin and ocular and rectal mucosa [99].

There were no specific studies found evaluating the expectorant and decongestant activities of *M. indica*. Overall, the antiviral and antibacterial activities of various parts of the plant support its use in URTI management.

#### 3.2.5. *Azadirachta indica* A.Juss. (Meliaceae)

*Azadirachta indica* (neem tree) is used in the following products: Muwereza herbal cough remedy®; Princess aloe lip balm®, a lip balm®; and Rezalin for ulcer®, a syrup for management of gastric ulcers, flatulence, and constipation. To support its use in a cough remedy, *A. indica* should possess expectorant, decongestant, antiviral, and/or antibacterial activities [82].

The bark extract of *A. indica* significantly blocked HSV-1 entry into cells at concentrations ranging from 50 to 100 *μ*g/ml [100]. The methanolic extract fraction of leaves of *A. indica* is virucidal against Coxsackie virus B-4 and interfered with an early event in the viral replication [101]. Thus, the antiviral activity has been demonstrated but not in the viruses known to commonly cause URTI, such as the rhinovirus, adenovirus, influenza virus, enterovirus, and respiratory syncytial virus.

The bark, leaf, seed, and fruit extracts of *A. indica* showed a concentration-dependent antibacterial activity against bacteria isolated from an adult mouth [102]. The ethanolic extract of neem twigs, barks, and leaves are bactericidal against *Streptococcus mutans* [103].

The methanol fraction of neem oil possesses bactericidal activity against 9 strains of *Helicobacter pylori*, the causative agent of gastric ulcers [104]. The extract's minimum inhibitory concentration (MIC) and minimum bactericidal concentration (MBC) against the *H. pylori* strains ranged between 25 and 51 *μ*g/ml and 43–68 *μ*g/ml, respectively. The bactericidal activity was time- and concentration-dependent [104]. This evidence supports the use of neem in Rezalin® syrup to manage ulcers.

The wound-healing effect of neem could be beneficial in the lip balm. The water extract of the stem bark of *A. indica* promoted wound healing in mice by increasing the rate of wound contraction and levels of hydroxyproline, DNA and protein content, and nitric oxide compared with the vehicle control group. These effects promote wound healing through increased inflammatory response and neovascularization [105].

Neem oil poisoning is rare in adults. However, one such case was reported in an adult male who presented with vomiting, seizures, metabolic acidosis, and toxic encephalopathy [106]. The patient had a complete recovery from symptomatic management. *In vivo* evaluation of acute toxicity of water extracts of *A. indica* leaves and seeds in rats revealed an LD_50_ of 6.2 and 9.4 ml/kg, respectively [107].

There is evidence to support the use of *A. indica* in URTI management, gastric ulcers associated with *H. pylori* infection, and wound healing. The use of the oil has the potential to cause adverse events.

#### 3.2.6. *Syzygium aromaticum* (L.) Merr. and L.M.Perry (Myrtaceae)

*S. aromaticum* (clove) was found in Kwesiima cough mixture® (a preparation for management of URTI) and Nana herbal mouth wash® (for management of toothache, tooth decay, sensitivity, bleeding gums). The antibacterial, anti-inflammatory, analgesic, anesthetic, and antiviral activities of *S. aromaticum* are important for these applications.

*S. pyogenes* and *S. mutans* are the most common causes of bacterial URTI and tooth decay, respectively [108]. The essential oil extracted from the dried flower buds of clove has antiseptic, anti-inflammatory, analgesic, and anesthetic activities [109]. This supports its common use in dentistry. Furthermore, eugenol, the main constituent of clove essential oil, showed an antinociceptive effect in a dose-dependent manner as measured in the acetic acid-induced writhing test in mice, with the effect lasting at least 30 minutes [110].

The methanolic extract of *S. aromaticum* has potent inhibitory activity against both dental caries pathogens with MIC of 0.2 mg/ml. In addition, *S. aromaticum* bud essential oil was active against *S. mutans* at 0.05 mg/ml [111]. Furthermore, the antibacterial activity of clove has been demonstrated in *S. aureus, Pseudomonas aeruginosa, E. coli*, *S. pyogenes*, *Corynebacterium species, Salmonella species,* and *Bacteroides fragilis* [112].

An *in vitro* study demonstrated cytotoxic properties of clove oil and eugenol toward human fibroblasts and endothelial cells. Clove oil was highly cytotoxic at concentrations as low as 0.03% (v/v) with up to 73% of this effect attributable to eugenol [113]. Moreover, eugenol produces local irritative and cytotoxic effects and hypersensitivity reactions when in contact with soft tissues [114]. However, Vijayasteltar et al. [115] evaluated the subchronic toxicity of the standardized polyphenolic extracts of clove buds in Wister rats and showed a no-observed-adverse-effect level at 1000 mg/kg bodyweight/day. This study showed that administration of the extracts did not result in any toxicologically significant changes in clinical/behavioral observations, ophthalmic examinations, body weights, organ weights, feed consumption, urinalysis, hematology, and clinical biochemistry parameters when compared with the untreated control group of animals. Also, no genotoxicity was observed with the extract rather exhibiting significant antimutagenic potential against the known mutagens: sodium azide, NPD, tobacco, and 2-acetamidoflourene.

The antibacterial, anti-inflammatory, analgesic, and anesthetic activities of *S. aromaticum* provide evidence for its value in managing URTI and dental conditions.

#### 3.2.7. *Aloe barbadensis* (L.) Burm.f. (Asphodelaceae)

*Aloe barbadensis* is a constituent of Nana herbal mouth wash®, Damaleo vera tablets® (for cleansing the GIT, promotion of kidney function, and boosting the immune system), Muwereza herbal cough remedy®, and Princess aloe lip balm®.

A wound dressing gel containing acemannan, extracted from the internal leaf aloe gel, reduced radiation-induced skin reactions in mice when applied daily for at least 2 weeks, beginning immediately after irradiation [116]. Furthermore, the extracts of crude *A. barbadensis* gel prevent suppressing the induction of T cell-mediated responses such as contact and delayed-type hypersensitivity caused by cutaneous exposure to ultraviolet light [117]. This effect is attributed to aloe oligosaccharides that prevent ultraviolet-induced suppression of delayed-type hypersensitivity by reducing keratinocyte-derived immunosuppressive cytokines such as interleukin-10. This evidence supports the use of aloe extracts in lip balm.

Acemannan from *A barbadensis* extract stimulated macrophage production of monokines, including interleukin-1 and tumor necrosis factor, resulting in the initiation of immune attack, necrosis, and regression of implanted sarcomas in mice [118]. In another study, *A. barbadensis* stimulated cellular and humoral immune responses in rabbits following immunization against myxomatosis [119]. Furthermore, aloe extracts enhanced the ability of broiler chickens to mount a humoral immune response when challenged with Newcastle disease virus [120]. These studies support the use of *A. barbadensis* extract to boost the immune system.

Emodin, an anthraquinone prepared from aloin, was shown to inactivate herpes simplex virus type 1 and type 2, varicella-zoster virus, pseudorabies virus, and influenza virus, not adenovirus and rhinovirus. Electron microscopic examination of the anthraquinone-treated herpes simplex virus demonstrated that the envelopes were partially disrupted [121]. This antiviral activity is important in the management of URTI.

Aloe emodin is also a known stimulant laxative [122], and this could be the basis of the use of *A. barbadensis* in Damaleo vera tablets for cleansing the GIT.

The generative changes in the kidney tissue were significantly reduced in diabetic rats treated with glibenclamide and aloe leaf gel and pulp extracts compared with the untreated group [123]. The treated group also had decreased serum urea and creatinine levels in comparison with diabetic controls. Among the diabetic rats, kidney lipid peroxidation levels were lower in those received aloe extracts than those who received glibenclamide alone. These findings suggest a protective effect of *A. barbadensis* on mild damage caused by type-II diabetes on kidney tissue and support its use in Damaleo vera tablets.

Several single case reports have been published on the toxicity of *A. barbadensis* in humans. Some include massive intraoperative bleeding after consumption of *A. barbadensis* tablets [124], acute renal failure [125], severe vomiting [126], acute hepatitis [127], and Henoch-Schonlein purpura [128]. Ernst [129] reported on the hypersensitivity and allergic reactions due to *A. barbadensis*. In another study, rats fed aloe whole-leaf powder for 90 days showed the adverse effect level to be 2 g/kg BW [130]. Aloe inner leaf gel rich in high-molecular-weight fibers and soluble polysaccharides is cytotoxic to the human intestinal cell line Caco-2 (CC_50_ = 1 g/l), even at concentrations below the recommended dose for human consumption [131]. Processing of aloe inner leaf gel or aloe leaf extract by filtration through diatomaceous earth and activated carbon reduces their cytotoxicity and improves their immunomodulatory activity [131].

Literature supports the use of aloe extracts for cosmetic purposes, boosting the immune system, and management of URTI and constipation. However, studies also show that aloe extracts can be toxic, although processing can reduce such toxicities.

#### 3.2.8. *Warburgia ugandensis* Sprague. (Canellaceae)

*W. ugandensis* is a constituent of herbal preparations including Lucas syrup®, Sacco syrup®, Focus herbal cough syrup® used in the management of URTI, and Jenacid negus® for the management of gastric ulcers.

The water extracts of *W. ugandensis* elicited antimicrobial activity against *E. coli* and *S. aureus* in the agar well assay, with higher activity against *S. aureus* than *E. coli.* This activity was only detectable at high concentrations of the extract, i.e., up to 50 mg/ml of crude extract [132]. In another study, the crude and petroleum ether extracts of *W. ugandensis* leaves exhibited antimicrobial activity against *S. boydii* and *S. aureus* [133]. The biological activities of the plant are attributed to the drimane and colorotane sesquiterpenoids, including polygodial, warburganal, muzigadial, mukaadial, and ugandensial, as well as flavonoids and other compounds [134, 135]. The sesquiterpene muzigadial isolated from the ethyl acetate extract of the stem bark of *W. ugandensis* was the main antibacterial agent (IC_50_ = 12.5 *μ*g/ml) against both *S. aureus* and *Bacillus subtilis* [136].

Specific antiviral activity studies could not be found to assess the importance of *W. ugandensis* in the management of viral URTI. Its usefulness in the management of peptic ulcer disease could also not be established from the literature review. Other reported pharmacological activities of *W. ugandensis* backed by some scientific evidence include antifungal activity, antimycobacterial activity, anti-inflammatory and antioxidant properties, molluscicidal, antifeedant, antiplasmodial, cytotoxic, anthelmintic, and antileishmanial activities [135]. The diethyl ether extract of *W. ugandensis* has anti-inflammatory effects and inhibited both COX-2 and COX-1 *in vitro* with IC_50_ values of 13.33 ± 4.36 and 11.05 ± 1.43 *μ*g/ml, respectively [137].

While *W. ugandensis* is widely used by herbalists in Uganda for treating various infections, it is also known to be toxic if used in high doses [80]. The acute toxicity evaluation of the aqueous stem bark extract of *W. ugandensis* in mice gave an LD_50_ >5000 mg/kg body weight with no mortalities recorded for all dose levels used. The extract was classified as noncytotoxic with 50% cytotoxic concentration (CC_50_) determined to be >250 *μ*g/ml [138]. *W. ugandensis* was cytotoxic to intestinal epithelial cells IEC-6, with IC_50_ values <50 *μ*g/ml [139]. Both the DMSO (CC_50_ = 1.5 *μ*g/ml) and the ethanol (CC_50_ = 7.6 *μ*g/ml) root were highly cytotoxic to U87CD4CXCR4 cells [140].

The evidence in the literature is not enough to draw conclusions on the usefulness of *W. ugandensis* in the management of diseases of the respiratory tract or gastric ulcers. It can be toxic when used in high doses.

#### 3.2.9. *Zingiber officinale* Roscoe (Zingiberaceae)

*Zingiber officinale* (ginger) is locally used as a constituent of syrups for the management of URTI including Yeco cough doctor® and Muwereza herbal cough remedy®.

*Z. officinale* contains various phytochemicals including phenolic compounds, terpenes, polysaccharides, lipids, organic acids, and raw fibers. The pharmacological activities of ginger have however been attributed to phenolic compounds such as gingerols and shogaols [141]. Various studies have demonstrated the anti-inflammatory activity of ginger and its bioactive compounds. Orally administered ginger reduced the proinflammatory cytokines (TNF-*α*, IL-6, and IL-1*β*) and increased the anti-inflammatory cytokines (IL-10 and IL-22) in mouse colitis models [142]. *Z. officinale* attenuated post-exercise-induced elevation in several proinflammatory cytokines including interleukin-1*β* (IL-1*β*), interleukin-6 (IL-6), and tumor necrosis factor-*α* (TNF-*α*) in well-trained male endurance runners [143].

Intravenous administration of 6-gingerol (at 1.75–3.5 mg/kg) and 6-shogaol (at 1.75–3.5 mg/kg) and oral administration of both (at 70–140 mg/kg) produced an inhibition of spontaneous motor activity, as well as antipyretic and analgesic effects, the latter producing more intense effects than the former. 6-Shogaol also showed an intense antitussive effect in comparison with dihydrocodeine phosphate [144]. This supports of the use of *Z. officinale* in herbal cough remedies for their cough-suppressant activity.

The ethanolic extract of *Z. officinale* rhizome was active against common respiratory tract pathogens: *S. aureus, S. pyogenes, S. pneumoniae*, and *Haemophilus influenzae* [145]. Furthermore, an herbal extract of *Z. officinale* inhibited biofilm formation and affected the membrane integrity of multidrug-resistant *P. aeruginosa*, both of which play a role in the mechanism of infection and antimicrobial resistance [146]. The crude and methanolic fraction of ginger reduced biofilm formation, inhibited glucan synthesis and adhesion, and downregulated the virulence genes in *S. mutans* [147].

The antiviral activity of ginger has been demonstrated in some viruses not specifically implicated in the causation of common URTI. Fresh *Z. officinale* dose-dependently inhibited human respiratory syncytial virus (HRSV) and induced plaque formation in human respiratory tract cell lines [148]. Fresh ginger also dose-dependently inhibited viral attachment and internalization and, at high concentration, stimulated mucosal cells to secrete interferon *β* (IFN-*β*) that possibly contributed to counteracting viral infection. Other studies have demonstrated antiviral activity of *Z. officinale* in hepatitis C virus (HCV) [149] and Chikungunya virus that causes an arthritogenic febrile illness [150].

Studies on the safety of different *Z. officinale* rhizome extracts showed no treatment-related signs of toxicity or mortality in any animals at tested doses [151–154]. One such study revealed a no-observed-adverse-effect level for a crude ethanolic extract of 5000 mg/kg body weight [151].

There is scientific evidence in support of the use of *Z. officinale* extracts in preparations for the management of upper respiratory tract diseases. The rhizome has been established to be safe.

#### 3.2.10. *Allium sativum* L. (Amaryllidaceae)

From the survey, *Allium sativum* (garlic) is used in Jenacid negus® for the management of peptic ulcer disease and in Yeco cough doctor® for the management of URTI.

An aqueous garlic extract was bactericidal against 16 clinical isolates and three reference strains of *Helicobacter pylori*. The MIC_90_ of the extracts was 5 mg/ml, and the MBC was equal to, or twofold higher than, the MIC. Boiling the garlic extract lowered the efficacy twofold to fourfold the values of MIC and the MBC obtained with fresh extract [155]. Another study in which the aqueous garlic extract was standardized for its thiosulfinate concentration revealed an MIC of 40 *μ*g/ml of thiosulfinate against *H. pylori* [156].

Orally administered *A. sativum* juice at 250 and 500 mg/kg in rats produced a gastric ulcer healing effect in acetic acid-induced chronic gastric ulcer. The extracts also produced a gastric antisecretory effect in pylorus-ligated rats; gastric cytoprotective effect in ethanol-induced and indomethacin-induced gastric ulcer; and a significant reduction in stress-induced gastric ulcers and cysteamine-induced duodenal ulcers with higher effectiveness at 250 mg/kg [157]. Furthermore, aqueous garlic extract increased the pH and reduced the volume of gastric juice in rats [158].

An ethanolic garlic extract was bactericidal against *S. pyogenes*, which is commonly implicated in bacterial URTI, with an MBC of 1 g/ml [159]. The antiviral activity of garlic has been demonstrated in the following viruses known to cause URTI in humans; adenovirus [160]; influenza A and influenza B virus [161, 162]; human rhinovirus-2 [163]; and measles virus, Newcastle disease virus, and parainfluenza virus-3 [164]. Garlic acts by blocking viral entry into host cells, inhibiting viral RNA polymerase, reverse transcriptase, DNA synthesis, and immediate-early gene 1 (IEG1) transcription, as well as through downregulating the extracellular-signal-regulated kinase (ERK)/mitogen-activated protein kinase (MAPK) signaling pathway [164]. These activities can be important in the management of URTI of different etiologies.

The most commonly reported adverse effects of garlic in randomized clinical trials are garlic breath and gastrointestinal symptoms [165]. Allergic reactions have also been reported in case reports [166–168]; contact dermatitis [169] and drug-herb interactions have been reported in patients taking anticoagulant medication (warfarin) and those on antiretroviral therapy [170].

Scientific evidence supports the use of *A. sativum* in the management of upper respiratory tract conditions and in peptic ulcer disease. The plant can be used with caution as cases of allergic reactions have been reported.

#### 3.2.11. *Eucalyptus globulus* Labill. (Myrtaceae)

*Eucalyptus globulus* is a constituent of Nana herbal mouth wash®, Princess pain balm®, Phycof cough syrup®, Focus herbal cough syrup®, Lucas syrup®, Kwesiima cough mixture®, Gabogola syrup®, and Sacco syrup®.

An essential oil blend of *E. globulus* with other oils was effective against fourteen Gram-positive and Gram-negative bacteria strains tested, including some antibiotic-resistant strains [171]. The organisms tested in the study included those implicated in causation of URTI; for example, *S. pyogenes*, *S. pneumoniae*, *Listeria monocytogenes*, and *S. aureus.* MICs ranged from 0.01% to 3%. The study also found the blend effective against H1N1 influenza virus, making it suitable for management of influenza and post-influenza bacterial pneumonia infections [171]. The antibacterial effect of *E. globulus* has also been demonstrated in the organisms causing dental caries especially *S. mutans* [172] and *Lactobacilli* [173, 174].

The essential oil of *E. globulus* exhibited analgesic and anti-inflammatory effects in mice and rat models [175]. The essential oil of *E. globulus* possesses central and peripheral analgesic effects as well as neutrophil-dependent and neutrophil-independent anti-inflammatory activities [175].

A eucalyptus oil water emulsion had an LD_50_ of 3811.5 mg/kg. The oil slowed growth of the male rats and damaged the liver and the kidneys at 792 and 1188 mg/kg BW after oral administration, respectively [176]. In another study, an LD_50_ of 1650 mg/kg in mice was determined. In addition, there was no abnormal skin reaction observed when a 5% ointment formulation was administered on the rats' skin, indicating the safety of *E. globulus* essential oil at a relatively lower concentration [177].

*E. globulus* is safe for use, at low concentrations, for the management of URTI and dental conditions.

Table 3 shows the evidence of safety and efficacy of the plant raw materials used to manufacture herbal products in Uganda.

### 3.3. Availability of Plant Monographs

We reviewed the WHO monographs, African Pharmacopoeia, and West African Pharmacopoeia to obtain information on the availability of monographs for the plants used to manufacture products in Uganda. Of the 33 plant materials, only ten (10) had monographs published by the WHO, ten (10) by the African Pharmacopoeia, and six (6) by the West African Pharmacopoeia. Nonetheless, *Aloe vera* (L.) Burm.f. (Asphodelaceae), *Azadirachta indica* A.Juss. (Meliaceae), *Zingiber officinalis* Roscoe (Zingiberaceae), and *Allium sativum* L. (Amaryllidaceae) had monographs published by all three authorities (Table 2). The folk and/or traditional uses in these pharmacopoeias are similar to the Ugandan manufacturers' as indicated on the product labels for most of the herbs. In addition to the traditional uses, the monographs reviewed also state the pharmacological evidence, plant parts of interest, methods for preparation, and quality control procedures. Therefore, these monographs can be adopted for routine quality control of the materials produced in Uganda.

Table 2 summarizes the availability of plant monographs in African Pharmacopoeia, West African Herbal Pharmacopoeia, and WHO monographs on selected herbal materials. The traditional indications are outlined as they are stated in the monographs for comparison with Ugandan manufacturer indications of the products containing the plant materials to justify adaptability of the monographs.

## 4. Conclusion

Several *in vitro* and *in vivo* studies have been carried out to establish the safety and efficacy of the plants used for the management of different conditions. For all plant materials, there is some scientific evidence to support the use of the plants in the management of the conditions on the label claim. However, some of these studies lack specificity for the conditions on the label claim and therefore may not be confidently relied upon to support the use of the plant material. More specific bioassays are required in these cases to provide conclusive evidence. Also, studies in humans are few, and therefore, in most cases, results from animal models and human cell lines are extrapolated to humans. Some plant materials have specific dose-dependent toxicities and interactions that should concern the manufacturers and the regulatory authorities before approval of the products. Other plants' toxicity depends on the preparation method, yet there are no GMP requirements for herbal manufacture in Uganda. Pharmacovigilance efforts should be stepped up to identify toxicity cases and establish efficacy of the finished products. Plant monographs are urgently required to standardize uses, processing, and quality assurance of plant materials.

## Figures and Tables

**Table 1:** Plant materials used for the manufacture of local herbal medical products in Uganda.

No.	Product	Indication(s) as per product label	Verified scientific names (family)
1	Nana herbal mouth wash®	Toothache	(i) *Calendula officinalis* L. (Asteraceae)
		Bad odor	(ii) *Krameria lappacea* (Dombey) Burdet and B.B. Simpson (Krameriaceae)
		Sensitivity	(iii) *Camellia sinensis* (L.) Kuntze (Theaceae)
		Bleeding gums	(iv) *Carica papaya* L. (Caricaceae)
		Cavities	(v) *Eucalyptus globulus* Labill. (Myrtaceae)
		Tooth decay	(vi) *Aloe vera* (L.) Burm.f. Synonym: *Aloe barbadensis* (Asphodelaceae)
		Antibacterial	(vii) *Terminalia catappa* L. (Combretaceae)
			(viii) *Salvadora persica* L. (Salvadoraceae)
			(ix) *Syzygium aromaticum* (L.) Merr. and L.M.Perry (Myrtaceae)
			(x) *Mentha* × *piperita* L. (Lamiaceae)

2	Gabogola syrup®	Whooping cough, catarrh	(i) *Mangifera indica* L. (Anacardiaceae)
		Sore throat	(ii) *Eucalyptus globulus* Labill. (Myrtaceae)
		Congestion from asthma and bronchitis	(iii) *Albizia coriaria* Oliv. (Fabaceae)

3	Kwesiima cough mixture®	Cough	(i) *Albizia coriaria* Oliv. (Fabaceae)
		Catarrh	(ii) *Mangifera indica* L. (Anacardiaceae)
		Sore throat	(iii) *Eucalyptus globulus* Labill. (Myrtaceae)
		Congestion from asthma and bronchitis	(iv) *Syzygium aromaticum* (L.) Merr. and L.M.Perry (Myrtaceae)

4	Lucas syrup®	Cough	(i) *Eucalyptus globulus* Labill. (Myrtaceae)
		Flu	(ii) *Warburgia ugandensis* Sprague (Canellaceae)
		Mouth sores	
		Measles symptoms	

5	Yeco cough doctor®	Allergic cough	(i) *Zanthoxylum chalybeum* Engl. (Rutaceae)
		Smokers cough	(ii) *Zingiber officinale* Roscoe (Zingiberaceae)
		Whooping cough	(iii) *Combretum molle* R.Br. ex G.Don (Combretaceae)
		Productive cough	(iv) *Morella kandtiana* (Engl.) Verdc. and Polhill (Myricaceae) Synonym: *Myrica kandtiana*
		Flu	(v) *Mangifera indica* L. (Anacardiaceae)
		Lung cleaning	(vi) *Allium sativum* L. (Amaryllidaceae)

6	Damaleo vera tablets®	Cleanses GIT	(i) *Aloe vera* (L.) Burm.f. (Asphodelaceae) Synonym: *Aloe barbadensis*
		Promotes kidney function	
		Boost immune system	

7	Muwereza herbal cough remedy	Cough	(i) *Azadirachta indica* A.Juss. (Meliaceae)
		Flu	(ii) *Aloe vera* (L.) Burm.f. (Asphodelaceae) Synonym: *Aloe barbadensis*
		Sore throat	(iii) *Zingiber officinale* Roscoe (Zingiberaceae)
		Sinusitis	(iv) *Dracaena steudneri Engl.* (Asparagaceae)
			(v*) Albizia coriaria* Oliv. (Fabaceae)

8	Rezalin for ulcer®	Gastric ulcers	(i) *Kigelia africana* (Lam.) Benth. (Bignoniaceae)
		Stomach ulcers	(ii) *Tamarindus indica* L. (Fabaceae)
		Flatulence	(iii) *Azadirachta indica* A.Juss. (Meliaceae)
		Constipation	(iv) *Entada abyssinica* A.Rich. (Fabaceae)
			(v) *Salvia hispanica* L. (Lamiaceae).

9	Sacco syrup®	Common colds	(i) *Morella kandtiana* (Engl.) Verdc. and Polhill (Myricaceae) Synonym: *Myrica kandtiana*
		Flu	(ii) *Eucalyptus globulus* Labill. (Myrtaceae)
		Cough	(iii) *Musa* × *paradisiaca* (Musaceae)
		Sinusitis	(iv) *Warburgia ugandensis* Sprague (Canellaceae)
		Rhinitis	
		Asthma	
		Catarrh	
		Whooping cough	
		Allergic conditions	

10	Replenish capsules®	Immune booster	(i) *Moringa oleifera* Lam. (Moringaceae)
		Antioxidant	

11	Focus herbal cough syrup®	Bronchial congestion	(i) *Warburgia ugandensis* Sprague (Canellaceae)
		Flu associated with cough	(ii) *Eucalyptus globulus* Labill. (Myrtaceae)
		Painful coughing	(iii) *Fragaria* × *ananassa* (Duchesne ex Weston) Duchesne ex Rozier (Rosaceae)
		Dry/irritating cough	
		Mouth sores	
		Hiccups	

12	Jenacid negus®	Ulcers	(i) *Zanthoxylum fagara* (L.) Sarg. (Rutaceae)
			(ii) *Solanum* spp. *(Solanaceae).*
			(iii) *Warbugia ugandensis* Sprague (Canellaceae)
			(iv) *Curcuma longa* L. (Zingiberaceae)
			(v) *Allium sativum* L. (Amaryllidaceae)

13	Witch hazel®	Relief of minor skin irritations due to insect bites	(i) *Hamamelis virginiana* L. (Hamamelidaceae)
		Minor cuts, minor scrapes	

14	Phycof cough syrup®	Dry, wet, and whooping cough	(i) *Zingiber officinale* Roscoe (Zingiberaceae)
		Sore throat	(ii) *Curcuma longa* L. (Zingiberaceae)
		Bronchial asthma	(iii) *Eucalyptus globulus* Labill. (Myrtaceae)
		Relieving fever	(iv) *Mentha × piperita* L. (Lamiaceae)
		Nasal congestion	(v) *Cymbopogon citratus* (DC.) Stapf (Poaceae)
			(vi) *Citrus limon* (L.) Osbeck
			(vii) *Tinospora sinensis* (Lour.) Merr. (Menispermaceae). Synonym: *Tinospora cordifolia*
			(viii) *Albizia coriaria* Oliv. (Fabaceae)
			(ix) *Acorus calamus* L. (Acoraceae)
			(x) *Xanthium strumarium* L. (Astaraceae)

15	Princess aloe lip balm®	Dry, cracked, and painful lips	(i) *Aloe vera* (L.) Burm.f. (Asphodelaceae) Synonym: *Aloe barbadensis*
			(ii) *Azadirachta indica* A.Juss. (Meliaceae)

16	Princess pain balm®	Pain relief	(i) *Eucalyptus globulus* Labill. (Myrtaceae)
			(ii) *Cinnamomum verum* J.Presl (Lauraceae) Synonym: *Cinnamomum zeylanicum*

**Table 2:** Availability of plant monographs in African- and WHO-published pharmacopoeia/monographs.

No.	Plant material (manufacturer use in Uganda)	African Pharmacopoeia, 2014 [15]	West African Herbal Pharmacopoeia, 2013 [16]	WHO monographs (vol I–IV)
		(i) Part(s) of plant used	(i) Part(s) of plant used	(i) Part(s) of plant used
		(ii–iv) Similar traditional use	(ii–iv) Similar traditional use	(ii–iv) Similar traditional use
1.	*Eucalyptus globulus* Labill.		No monograph	
	(i) Mouth wash	(i) Oil		(i) Oil
	(ii) URT disorders	(ii) Astringent, antiseptic		(ii) Dental caries, ulcers of the skin
	(iii) Analgesic	(iii) Asthma		(iii) Catarrh and coughs, sinusitis

2.	*Aloe vera (L.)* Burm.f.	(i) Dried juice	(i) Decoction, juice, dried juice	(i) Dried juice, gel
	(i) Wound healing		(ii) Dermatitis, thermal and sunburns	
	(ii) Mouth wash		(iii) Cold	(ii) Seborrhoeic dermatitis, minor wounds and inflammatory skin disorders, haemorrhoids
	(iii) Immune booster	(ii) Cathartic	(iv) Peptic ulcer	(iii) Peptic ulcers
	(iv) GIT disorders			
	(v) Promote kidney function			

3.	*Albizia coriaria* Oliv.	No monograph	No monograph	No monograph
4.	*Mangifera indica* L.	No monograph	No monograph	No monograph
5.	*Warburgia ugandensis* Sprague	No monograph	No monograph	No monograph

6.	*Azadirachta indica* A.Juss.	(i) Leaves	(i) Leaves, stem bark, seeds	(i) Leaves
	(i) URT disorders,		(ii) Cough,	(ii) Asthma
	(ii) Wound healing	(ii) Inflammatory agent	(iii) Skin disorders, boils, ulcers, eczema	(iii) Wounds, bruises, allergic skin itching due to varicella, psoriasis, scabies, smallpox, warts

7.	*Zingiber officinale* Roscoe	(i) Rhizome	(i) Rhizome	(i) Rhizome
	(i) URT disorders	(ii) No similar traditional use	(ii) cough, colds, flu, asthma	(ii) Cold and flu, anti-inflammatory agent

8.	*Allium sativum* L.	(i) Bulb	(i) Bulb, oil from bulb	(i) Bulb
	(i) GIT disorders (peptic ulcers)	(ii) Antimicrobial	(ii) Antidiarrheal, stomachic, dysentery	(ii) Dysentery, ulcers, carminative, cholera, colic
	(ii) URT disorders		(iii) Expectorant, broad spectrum antibiotic	(iii) Asthma, expectorant, bronchitis

9.	*Syzygium aromaticum* (L.) Merr. and L.M.Perry	(i) Flower buds, oil	No monograph	(i) Flower buds
	(i) Mouth wash	(ii) Toothache		(ii) Mouthwashes, treatment of toothache, bleeding gums, and minor infections of the mouth
	(ii) URT disorders			(iii) Sore throats and coughs associated with the common cold, asthma

10.	*Morella kandtiana* (Engl.) Verdc. and Polhill	No monograph	No monograph	No monograph

11.	*Curcuma longa* L.	No monograph	No monograph	(i) rhizome
	(i) GIT disorders (Gastric ulcers)			(ii) Peptic ulcers, diarrhea
	(ii) URT disorders			(iii) Coughs

12.	*Mentha piperita* L.	(i) Leaves, oil	No monograph	(i) Essential oil
	(i) URT disorders	No similar traditional use		(ii) Symptomatic treatment of catarrh and coughs
	(ii) Mouth wash			

13.	*Moringa oleifera* Lam.	(i) Seeds	(i) Leaves, flower, fruit, root, seed	No monograph
	(i) Immune booster	(ii) Wound healing,	(ii) Supplements	
	(ii) Antioxidant	(iii) Antioxidant, anti-inflammatory		

14.	*Carica papaya* L.	(i) Fresh fruits	(i) Leaf, fruit or root, seed	No monograph
	(i) Mouth wash	(ii) GIT disorders	(ii) Dental caries	
15.	*Terminalia catappa* L.	No monograph	No monograph	No monograph
16.	*Salvadora persica*	No monograph	No monograph	No monograph
17.	*Zanthoxylum fagara* (L.) Sarg.	No monograph	No monograph	No monograph
18.	*Solanum* spp.	No monograph	No monograph	No monograph
19.	*Acorus calamus* L.	No monograph	No monograph	No monograph
20.	*Xanthium strumarium* L.	No monograph	No monograph	No monograph

21.	*Hamamelis virginiana* L.	No monograph	No monograph	(i) Leaves and bark
	(i) Skin ulcers,			(ii) Neuralgia, nosebleeds
	(ii) Skin irritations			

22.	*Calendula officinalis* L.	No monograph	No monograph	(i) Ligulate florets and composite flowers
	(i) Mouth wash			(ii) Superficial cuts, minor inflammations of the skin and oral mucosa, gastritis.
23.	*Zanthoxylum chalybeum* Engl.	No monograph	No monograph	No monograph
24.	*Combretum molle* R.Br. ex G.Don	No monograph	No monograph	No monograph
25.	*Dracaena steudneri* Engl.	No monograph	No monograph	No monograph
26.	*Kigelia africana* (Lam.) Benth.	No monograph	No monograph	No monograph

27.	*Tamarindus indica* L.	(i) Fruits and seeds	No monograph	No monograph
	(i) GIT disorders	(ii) Laxative		
28.	*Entada abyssinica* A.Rich.	No monograph	No monograph	No monograph
29.	*Salvia hispanica* L.	No monograph	No monograph	No monograph
30.	*Musa paradisiaca*	No monograph	No monograph	No monograph

31.	*Cymbopogon citratus* (DC.) Stapf.	(i) Herb	(i) Leaf, flower	No monograph
	(i) URT disorders	(ii) No similar traditional use	(ii) Anticatarrhal and antirheumatic	

32.	*Citrus limon* (L.) Osbeck	Plant called *Citrus limonum Risso*	No monograph	No monograph
		(i) Pericarp (fresh or dry)		
	(i) URT disorders	(ii) No similar traditional use		

33.	*Tinospora sinensis* (lour.) Merr.	No monograph	No monograph	No monograph

**Table 3:** Evidence of efficacy and safety for herbal materials used for manufacture of medicinal products in Uganda.

No.	Plant material	Use of product (containing the material) according to the label	Evidence of efficacy related to product label claim; mechanism of action	Evidence of safety
10.	*Morella kandtiana* (Engl.) Verdc. and Polhill	URT disorders	Root extracts exhibited low inhibitory effects on the growth of *Acinetobacter baumannii* CDC-0033 (IC_50_: 128 *μ*g/ml; MIC: >256 *μ*g/ml) and moderate effects on *Pseudomonas aeruginosa* AH-71 (IC_50_: 32 *μ*g/ml; MIC: 256 *μ*g/ml) [21]	Very low toxicity to human HaCaT cells IC_50_ = 512 *μ*g/ml [21].

11.	*Curcuma longa* L.	Gastric ulcers	*C. longa* extract inhibits gastric acid secretion by competitively blocking H(2) histamine receptors [22]	The extract was safe and efficacious in the treatment of painful knee osteoarthritis in a randomized placebo-controlled trial [23].
		URT disorders	Curcumin reduced ethanol-induced gastric lesions and significantly increased gastric blood flow and plasma gastrin levels in a dose-dependent manner. This involves endogenous PG, NO, gastrin, and CGRP released from sensory nerves due to activation of the vanilloid TRPV1 receptor. This protective effect can be attributed to the inhibition of HIF-1a and Cdx-2 expression and the activation of HO-1 and SOD 2 expression [24]	

12.	*Mentha piperita* L.	URT disorders	A spray containing essential oils of five plants including *M. piperita* caused significant and immediate improvement in symptoms of upper respiratory ailment in a randomized clinical trial [25]	Short-term and subchronic oral studies reported cyst-like lesions in the cerebellum in rats given high doses of peppermint oil (>200 mg/kg/day). Peppermint oil contains the hepatotoxin pulegone. Isolated clinical cases of irritation and/or sensitization to peppermint oil and/or its constituents have been reported [26].
		Mouth wash		

13.	*Moringa oleifera* Lam.	Immune booster	The methanolic leaf extract caused significant immune-stimulatory effects on both cell-mediated and humoral immune systems in the Wistar albino rats [27]. The ethanolic and saline extracts contain antioxidants [28]. The leaf extract increased the antioxidant activity of glutathione (186%), superoxide dismutase (97.8%) and catalase (0.177%) [29].	The leaves are genotoxic at supra-supplementation levels of 3000 mg/kg BW but are safe at levels ≤1000 mg/kg BW in rats (LD_50_ = 1585 mg/kg) and thus nontoxic [30].
		Antioxidant		

14.	*Carica papaya* L.	Mouth wash	A randomized, single-blind parallel-design study showed the leaf extract dentifrice/mouthwash provided an efficacious and natural alternative to sodium lauryl sulfate-free dentifrice ± essential oil-containing mouthwash in reducing interdental gingival inflammation [31]	The leaf extract is safe as shown in subacute oral toxicity tests in Sprague Dawley rats at up to 2 g/kg, (14 times the levels used in traditional medicine in Malaysia [32, 33]). Short-term leaf consumption in adults is generally safe though cautioned in pregnancy and liver impairment [34]

15.	*Terminalia catappa* L.	Mouth wash	The leaf aqueous and methanol extract demonstrated antibacterial activity against different bacterial strains [35]	The aqueous leaf extract is relatively nontoxic to the heart in subacute toxicity studies [36]

16.	*Salvadora persica* L.	Mouth wash	*S. persica* extract caused significant reduction in the plaque score and cariogenic bacterial count, but the score was lower than the positive control chlorhexidine mouthwash [37].	The aqueous and ethanolic extracts were not toxic to mice at doses of up to 1200 mg/kg [38]

17.	*Zanthoxylum fagara* (L.) Sarg	Gastric ulcers	No specific data for *Z. fagara.*	No specific data for *Z. fagara*

18.	*Solanum* spp.	Gastric ulcers	Methanolic, alcoholic, and aqueous extracts of *Solanum* species increase gastric mucus secretion and reduce HCl secretion [39], thus acting as a mechanical barrier and protecting the mucosa [40]	*Solanum* species contain toxic glycoalkaloids including solanine, chaconine, and solasodine. Toxicity manifests as gastroenteritis, weakness, excessive salivation, dyspnea, tremors, paralysis, prostration, and death [41]. Boiling reduces toxicity.

19.	*Acorus calamus* L.	URT disorders	Showed antibacterial activity against *Enterobacter aerogenes* and *S. aureus* and *Proteus mirabilis* [42].	The hydroalcoholic extract of *A. calamus* is nontoxic but at high dose produces mild but acceptable toxicity potential [43]

20.	*Xanthium strumarium* L.	URT disorders	Anti-inflammatory activity through inhibition of IFN-*γ* lipopolysaccharide-induced NO production and TNF-*α* production and analgesic activity [44] and inhibits histamine, interleukins, and TNF *α*, which are important in allergic rhinitis [45, 46],	A decoction caused hepatic injury, symptomatic hypoglycemia, and seizures 7 days after administration [47]. Extracts can induce *in vitro* DNA damage at cytotoxic concentrations *in vitro* [48] *X. strumarium* contains the toxic compounds atractyloside, carboxyatractyloside, and 4′-desulphate-atractyloside, which XSF induced significant cytotoxic effects in both L-02 and BRL liver cell lines [49]

21.	*Hamamelis virginiana* L.	Skin ulcers	A shampoo from *H. virginiana* improved subjective manifestations of skin irritation [50]. Extracts have antifungal and antibacterial effects against *Candida albicans* and *S. aureus* [51]	Did not cause any toxicity or deaths in rabbits when administered through the rectum at 300 mg/kg for 28 days [52]
		Skin irritations		

22.	*Calendula officinalis* L.	Mouth wash	A randomized controlled clinical study showed that a flower extract mouthwash of *C. officinalis* could be effective on reducing the intensity of radiation-induced oropharyngeal mucositis in patients with head-and-neck cancer [53]. In another randomized controlled study, *C. officinalis* mouth wash was shown to be effective in treating gingivitis by significantly reducing the plaque and gingival index [54]	Acute and subchronic toxicity studies indicated that the extracts of *C. officinalis* have low toxicities in Wistar rats [55]

23.	*Zanthoxylum chalybeum* Engl.	URT disorders	The essential oil exhibited strong antibacterial activities against *S. typhi, S. agalactia, S. aureus, and E. coli* [56]	The ethanol extract was weakly cytotoxic (CC_50_ = 231.0 *μ*g/ml), whereas the DMSO extract was moderately cytotoxic (CC_50_ = 39.8 *μ*g/ml) to U87CD4CXCR4 cells [57]. The methanol, ethanol, ethyl acetate, and diethyl ether extracts were not toxic to human keratinocyte cell line (HaCaT) IC_50_ >512 [21]

24.	*Combretum molle* R.Br. ex G.Don	URT disorders	*C. molle* has antibacterial activity. The MIC and MBC values ranged from 2.5 mg/ml to 5.0 mg/ml [58]	The aqueous leaf extract is moderately toxic when given to rats intraperitoneally at doses up to 8000 mg/kg [59].

25.	*Dracaena steudneri* Engl.	URT disorders	The leaves contain flavones and flavanones that inhibits proinflammatory cytokine release [60]	No specific data available

26.	*Kigelia africana* (Lam.) Benth.	Gastric disorders (ulcers, flatulence, constipation)	The aqueous leaf possesses antiulcer potential with protective and curative effects against gastric lesion [61]. *K. africana* at a concentration of 450 mg/kg BW gave the best results with a significant decrease in ulcer index (0.67 ± 0.16) on aspirin-induced ulcerogenic animals compared with 3.0 for the reference drug (cimetidine at 300 mg/kg) and control with 1.67 ± 0.27 [62]	The aqueous bark extract was safe in rats up to 5 g/kg [63]. At a higher dose of 6400, the extract was toxic in rats [64]

27.	*Tamarindus indica* L.	Gastric disorders (ulcers, flatulence, constipation)	The seed extract showed a dose-dependent protective effect on animal peptic ulcer models [65]. The leaf extract exhibited antiulcerogenic and ulcer-healing properties in rats [66].	Even at high concentrations, the extract administered to rats was still safe compared with the 5% concentration usually present in juice consumed by humans [67]

28.	*Entada abyssinica* A.Rich.	Gastric disorders (ulcers, flatulence, constipation)	Contains entadanin, which possess strong antibacterial activity against *Salmonella typhimurium* with the lowest MIC of 1.56–3.12 *μ*g/ml [68]	The IC_50_ values for the different active compounds ranged from 0.48 to 2.87 *μ*g/ml in the DPPH assay, from 2.53 to 17.04 *μ*g/ml in the ABTS assay, and from 1.43 to 103.98 *μ*g/ml in the FRAP assay. The compounds were less toxic than the positive control (LC_50_ values 22.42 to 80.55 *μ*g/ml) in Vero cells, suggesting relative lack of cytotoxicity [68].

29.	*Salvia hispanica* L.	Gastric disorders (ulcers, flatulence, constipation)	Chia seeds caused a significant decrease in symptoms of the constipation symptoms in the third week of treatment [69]	Chia seeds are safe as a novel food [70]

30.	*Musa paradisiaca*	URT disorders	Both the ethanol aqueous extracts showed antibacterial activity against *S. aureus*, *Vibrio cholerae, Shigella dysenteriae, Bacillus subtilis*, and *P. aeruginosa* (MIC of aqueous fraction = 3.125–25 mg/ml) [71]	The aqueous extract was relatively toxic in the acute toxicity test in Swiss Albino mice (LD_50_ = 489.9 mg/kg BW) [71]

31.	*Cymbopogon citratus* (DC.) Stapf	URT disorders	The oils are effective against *S. aureus*, *Bacillus cereus*, *Bacillus subtilis*, *Klebsiella pneumoniae*, and *E. coli* (MIC 0.03–0.5 mg/ml) [72]	The oils were nontoxic to rats. They did not induce any adverse effects to the blood, liver function, kidney function, protein, carbohydrate, and lipid metabolism [73]

32.	*Citrus limon* (L.) Osbeck	URT disorders	The peel extract exhibited potent antibacterial effects against *S. aureus, B. subtilis, E. coli, K. pneumonia*, and, *Salmonella typhi* (MIC = 50–6.25 mg/ml) [74]	Essential oils are generally nontoxic and have classified as generally recognized as safe (GRAS) by the FDA [75]

33.	*Tinospora sinensis* (Lour.) Merr.	URT disorders	Antioxidant [76], immunostimulatory, analgesic, anti-inflammatory, and antimicrobial activities against *S. aureus* [77, 78]	No toxicity data available

## Data Availability

The data sets analyzed during the current study are available from the corresponding author on reasonable request.

## References

[1] Tilburt J. C., Kaptchuk T. J. (2008). Herbal medicine research and global health: an ethical analysis. *Bulletin of the World Health Organization*.

[2] World Health Organization (2019). *WHO Global Report on Traditional and Complementary Medicine 2019*.

[3] Chan K. (2003). Some aspects of toxic contaminants in herbal medicines. *Chemosphere*.

[4] Anquez-Traxler C. (2011). The legal and regulatory framework of herbal medicinal products in the European Union: a focus on the traditional herbal medicines category. *Drug Information Journal*.

[5] Wu C., Lee S.-L., Taylor C. (2020). Scientific and regulatory approach to botanical drug development: a US FDA Perspective. *Journal of Natural Products*.

[6] World Health Organization (2005). *National Policy on Traditional Medicine and Regulation of Herbal Medicines: Report of a WHO Global Survey*.

[7] World Health Organization (2004). *Guidelines for Registration of Traditional Medicines in the WHO African Region*.

[8] World Health Organization (2007). *WHO Guidelines for Assessing Quality of Herbal Medicines with Reference to Contaminants and Residues*.

[9] World Health Organization (2007). *WHO Guidelines on Good Manufacturing Practices (GMP) for Herbal Medicines*.

[10] National Drug Authority (2021). *Drug Register*.

[11] National Drug Authority (2016). *Amateeka Agafuga Eddagala Lyekinnansi Mu Uganda*.

[12] Abidullah S., Rauf A., Zaman W. (2021). Consumption of wild food plants among tribal communities of Pak-Afghan border, near Bajaur, Pakistan. *Acta Ecologica Sinica*.

[13] Birjees M., Ahmad M., Zafar M. (2021). Traditional knowledge of wild medicinal plants used by the inhabitants of Garam Chashma valley, district Chitral, Pakistan. *Acta Ecologica Sinica*.

[14] Zhou J., Ouedraogo M., Qu F., Duez P. (2013). Potential genotoxicity of traditional Chinese medicinal plants and phytochemicals: an overview. *Phytotherapy Research*.

[15] Inter African Committee on Medicinal, African Traditional P. M., Organization of African U., Scientific T., Research C. (2014). *African Pharmacopoeia*.

[16] West African Health Organization (2013). *West African Herbal Pharmacopoeia BOBO-DIOULASSO (BURKINA FASO)*.

[17] Murray V., Shaw D. (2000). WHO monographs on selected medicinal plants, volume 2. *Health and Hygiene*.

[18] World Health Organization (1999). *WHO Monographs on Selected Medicinal Plants*.

[19] World Health Organization (2007). *WHO Monographs on Selected Medicinal Plants*.

[20] World Health Organization (2009). *WHO Monographs on Selected Medicinal Plants*.

[21] Schultz F., Anywar G., Tang H. (2020). Targeting ESKAPE pathogens with anti-infective medicinal plants from the Greater Mpigi region in Uganda. *Scientific Reports*.

[22] Kim D.-C., Kim S.-H., Choi B.-H. (2005). Curcuma longa extract protects against gastric ulcers by blocking H2 histamine receptors. *Biological and Pharmaceutical Bulletin*.

[23] Madhu K., Chanda K., Saji M. (2013). Safety and efficacy of *Curcuma longa* extract in the treatment of painful knee osteoarthritis: a randomized placebo-controlled trial. *Inflammopharmacology*.

[24] Czekaj R., Majka J., Magierowska K. (2018). Mechanisms of curcumin-induced gastroprotection against ethanol-induced gastric mucosal lesions. *Journal of Gastroenterology*.

[25] Ben-Arye E., Dudai N., Eini A., Torem M., Schiff E., Rakover Y. (2011). Treatment of upper respiratory tract infections in primary care: a randomized study using aromatic herbs. *Evidence-based Complementary and Alternative Medicine*.

[26] Nair B. (2001). Final report on the safety assessment of *Mentha piperita* (peppermint) oil, *Mentha piperita* (peppermint) leaf extract, *Mentha piperita* (peppermint) leaf, and *Mentha piperita* (peppermint) leaf water. *International Journal of Toxicology*.

[27] Nfambi J., Bbosa G. S., Sembajwe L. F., Gakunga J., Kasolo J. N. (2015). Immunomodulatory activity of methanolic leaf extract of *Moringa oleifera* in Wistar albino rats. *Journal of Basic and Clinical Physiology and Pharmacology*.

[28] Santos A. F., Argolo A. C., Paiva P. M., Coelho L. C. (2012). Antioxidant activity of *Moringa oleifera* tissue extracts. *Phytotherapy Research*.

[29] Moyo B., Oyedemi S., Masika P., Muchenje V. (2012). Polyphenolic content and antioxidant properties of *Moringa oleifera* leaf extracts and enzymatic activity of liver from goats supplemented with *Moringa oleifera* leaves/sunflower seed cake. *Meat Science*.

[30] Asare G. A., Gyan B., Bugyei K. (2012). Toxicity potentials of the nutraceutical Moringa oleifera at supra-supplementation levels. *Journal of Ethnopharmacology*.

[31] Saliasi I., Llodra J. C., Bravo M. (2018). Effect of a toothpaste/mouthwash containing *Carica papaya* leaf extract on interdental gingival bleeding: a randomized controlled trial. *International Journal of Environmental Research and Public Health*.

[32] Afzan A., Abdullah N. R., Halim S. Z. (2012). Repeated dose 28-days oral toxicity study of *Carica papaya* L. leaf extract in Sprague Dawley rats. *Molecules*.

[33] Ismail Z., Halim S. Z., Abdullah N. R., Afzan A., Abdul Rashid B. A., Jantan I. (2014). Safety evaluation of oral toxicity of *Carica papaya* Linn. leaves: a subchronic toxicity study in sprague dawley rats. *Evidence-based Complementary and Alternative Medicine*.

[34] Lim X., Chan J., Japri N., Lee J., Tan T. (2021). *Carica papaya* L. Leaf: a systematic scoping review on biological safety and herb-drug interactions. *Evidence-based Complementary and Alternative Medicine*.

[35] Nair R., Chanda S. (2008). Antimicrobial activity of *Terminalia catappa*, *Manilkara zapota* and *Piper betel* leaf extract. *Indian Journal of Pharmaceutical Sciences*.

[36] Iheagwam F. N., Okeke C. O., DeCampos O. C., Okere D. U., Ogunlana O. O., Chinedu S. N. (2019). Safety evaluation of *Terminalia catappa* Linn (Combretaceae) aqueous leaf extract: sub-acute cardio-toxicopathological studies in albino Wistar rats. *Journal of Physics: Conference Series, IOP Publishing*.

[37] Jassoma E., Baeesa L., Sabbagh H. (2019). The antiplaque/anticariogenic efficacy of Salvadora persica (Miswak) mouthrinse in comparison to that of chlorhexidine: a systematic review and meta-analysis. *BMC Oral Health*.

[38] Ezmirly S., Cheng J., Wilson S. (1979). Saudi Arabian medicinal plants: *Salvadora persica*. *Planta Medica*.

[39] Nguelefack T. B., Feumebo C. B., Ateufack G. (2008). Anti-ulcerogenic properties of the aqueous and methanol extracts from the leaves of *Solanum torvum* Swartz (Solanaceae) in rats. *Journal of Ethnopharmacology*.

[40] Saravanan S., Dhasarathan P., Indira V., Venkatraman R. (2011). Gastro protective and antioxidant activity of *Solanum nigrum* Linn. against aspirin and cold restraint stress induced ulcerated rats. *Research Journal of Immunology*.

[41] Slanina P. (1990). Solanine (glycoalkaloids) in potatoes: toxicological evaluation. *Food and Chemical Toxicology*.

[42] Mickymaray S., Al Aboody M. S. (2019). In vitro antioxidant and bactericidal efficacy of 15 common spices: novel therapeutics for urinary tract infections?. *Medicina*.

[43] Muthuraman A., Singh N. (2012). Acute and sub-acute oral toxicity profile of *Acorus calamus* (sweet flag) in rodents. *Asian Pacific Journal of Tropical Biomedicine*.

[44] An H.-J., Jeong H.-J., Lee E.-H. (2004). Xanthii fructus inhibits inflammatory responses in LPS-stimulated mouse peritoneal macrophages. *Inflammation*.

[45] Hong S.-H., Jeong H.-J., Kim H.-M. (2003). Inhibitory effects of *Xanthii fructus* extract on mast cell-mediated allergic reaction in murine model. *Journal of Ethnopharmacology*.

[46] Hong S.-H., Oh M.-J., Lee E.-J. (2004). Processed *Xanthii fructus* increases cell viability of mast cell line, RBL-2H3. *Oriental Pharmacy and Experimental Medicine*.

[47] Saidi H., Mofidi M. (2009). *Toxic Effect of Xanthium strumarium as an Herbal Medicine Preparation*.

[48] Piloto Ferrer J., Cozzi R., Cornetta T. (2014). *Xanthium strumarium* L. extracts produce DNA damage mediated by cytotoxicity in in vitro assays but does not induce micronucleus in mice. *BioMed Research International*.

[49] Xue L.-M., Zhang Q.-Y., Han P. (2014). Hepatotoxic constituents and toxicological mechanism of *Xanthium strumarium* L. fruits. *Journal of Ethnopharmacology*.

[50] Trüeb R. M. (2014). North American virginian witch hazel (*Hamamelis virginiana*): based scalp care and protection for sensitive scalp, red scalp, and scalp burn-out. *International Journal of Trichology*.

[51] Abbas T. F., Abbas M. F., Lafta A. J. (2020). Antibacterial activity and medical properties of Witch Hazel *Hamamelis virginiana*. *Annals of Tropical Medicine and Public Health*.

[52] Qinna N. A. (2013). Safety profile of suppository Hamamelis virginiana leaf extract. *Journal of Medicinal Plants Research*.

[53] Babaee N, Moslemi D, Khalilpour M (2013). Antioxidant capacity of calendula officinalis flowers extract and prevention of radiation induced oropharyngeal mucositis in patients with head and neck cancers: a randomized controlled clinical study. *Daru Journal of Pharmaceutical Sciences*.

[54] Khairnar M. S., Pawar B. W., Marawar P. P., Mani A. (2013). Evaluation of Calendula officinalis as an anti-plaque and anti-gingivitis agent. *Journal of Indian Society of Periodontology*.

[55] Lagarto A., Bueno V., Guerra I., Valdés O., Vega Y., Torres L. (2011). Acute and subchronic oral toxicities of *Calendula officinalis* extract in Wistar rats. *Experimental & Toxicologic Pathology*.

[56] Tigneh T. M., Neelaiah B. (2020). Variation in chemical composition and antimicrobial activities of essential oil of leaves of knob wood, *Zanthoxylum chalybeum* collected from three different places of eastern Ethiopia. *Oriental Journal of Chemistry*.

[57] Anywar G., Kakudidi E., Byamukama R., Mukonzo J., Schubert A., Oryem-Origa H. (2020). Data on medicinal plants used by herbalists for boosting immunity in people living with HIV/AIDS in Uganda. *Data in Brief*.

[58] Saidu T., Abdullahi M. (2011). Phytochemical determinations and antibacterial activities of the leaf extracts of *Combretum molle* and *Gossypium arboretum*. *Bayero Journal of Pure and Applied Sciences*.

[59] Dodehe Y., Rita B., Bernard N. D., N’guessan J. D. (2012). Acute and subacute toxic study of aqueous leaf extract of combretum molle. *Tropical Journal of Pharmaceutical Research*.

[60] Nchiozem-Ngnitedem V.-A., Omosa L. K., Bedane K. G., Derese S., Spiteller M. (2021). Inhibition of proinflammatory cytokine release by flavones and flavanones from the leaves of *Dracaena steudneri* engl. *Planta Medica*.

[61] Dos Santos M. M., Olaleye M. T., Ineu R. P. (2014). Antioxidant and antiulcer potential of aqueous leaf extract of Kigelia africana against ethanol-induced ulcer in rats. *EXCLI journal*.

[62] Orole R., Orole O., Adejumo T. (2013). Antiulcerogenic activity of *Kigelia africana*, *Nauclea latifolia* and *Staudtia stipitata* on induce ulcer in albino rats. *European Journal of Medicinal Plants*.

[63] Sharma U. K., Sharma U. S., Singh A., Agarwal V. (2010). Diuretic activity of *Kigelia pinnata* bark extract.

[64] Azu O. O., Duru F. I. O., Osinubi A. A. (2010). Histomorphometric effects of Kigelia africana (Bignoniaceae) fruit extract on the testis following short-term treatment with cisplatin in male Sprague-Dawley rats. *Middle East Fertility Society Journal*.

[65] Kalra P., Sharma S., Suman S. K. (2011). Antiulcer effect of the methanolic extract of *Tamarindus indica* seeds in different experimental models. *Journal of Pharmacy and Bioallied Sciences*.

[66] Raja N. L., Jegan N., Wesley J. (2008). Antiulcerogenic activity of alcoholic extract of the leaves of *Tamarindus indica* (L) on experimental ulcer models. *Pharmacologyonline*.

[67] Martinello F., Soares S., Franco J. (2006). Hypolipemic and antioxidant activities from *Tamarindus indica* L. pulp fruit extract in hypercholesterolemic hamsters. *Food and Chemical Toxicology*.

[68] Dzoyem J. P., Melong R., Tsamo A. T. (2017). Cytotoxicity, antimicrobial and antioxidant activity of eight compounds isolated from *Entada abyssinica* (Fabaceae). *BMC Research Notes*.

[69] Bernal Altamirano E., Iñaguazo Trávez J. J., Chanducas Lozano B. (2014). Effect of consumption of chia (*Salvia hispanica*) on constipation symptoms presented by students from a private university in Lima Este. *Revista Cient´ıfica de Ciencias de la Salud*.

[70] Turck D., Castenmiller J., De Henauw S. (2020). Scientific opinion on the safety of chia seeds (*Salvia hispanica* L.) subject to thermal processing in relation to the formation of process contaminants as a novel food for extended uses. *EFSA Journal*.

[71] Asuquo E. G., Udobi C. E. (2016). Antibacterial and toxicity studies of the ethanol extract of Musa paradisiaca leaf. *Cogent Biology*.

[72] Naik M. I., Fomda B. A., Jaykumar E., Bhat J. A. (2010). Antibacterial activity of lemongrass (*Cymbopogon citratus*) oil against some selected pathogenic bacterias. *Asian Pacific Journal of Tropical Medicine*.

[73] Mishra A., Kishore N., Dubey N., Chansouria J. (1992). An evaluation of the toxicity of the oils of *Cymbopogon citratus* and *Citrus medica* in rats. *Phytotherapy Research*.

[74] Kumar K. A., Narayani M., Subanthini A., Jayakumar M. (2011). Antimicrobial activity and phytochemical analysis of citrus fruit peels-utilization of fruit waste. *International Journal of Engineering Science and Technology*.

[75] Tisserand R., Young R. (2013). *Essential Oil Safety-E-Book: A Guide for Health Care Professionals*.

[76] Jain S., Sherlekar B., Barik R. (2010). Evaluation of antioxidant potential of *Tinospora cordifolia* and *Tinospora sinensis*. *International Journal of Pharmaceutical Sciences and Research*.

[77] Punitha D., Udhayasankar M., Danya U., Arumugasamy K., Shalimol A. (2013). Anti-inflammatory activity of characterized compound diosgenin isolated from *Tinospora malabarica* Miers in Ann.(Menispermaceae) in animal model. *International Journal of Herbal Medicine*.

[78] Sandhyarani G., Praveen Kumar K. (2014). Evaluation of analgesic activity of ethanolic extract of *Tinospora sinensis* leaves in rats. *International Journal of Preclinical and Pharmaceutical Research*.

[79] World Health Organization (2004). *Guidelines for Clinical Study of Traditional Medicines in WHO African Region*.

[80] Anywar G., Kakudidi E., Byamukama R., Mukonzo J., Schubert A., Oryem-Origa H. (2020). Indigenous traditional knowledge of medicinal plants used by herbalists in treating opportunistic infections among people living with HIV/AIDS in Uganda. *Journal of Ethnopharmacology*.

[81] Thomas M., Bomar P. A. (2020). *Upper Respiratory Tract Infection*.

[82] Irwin R. S., Baumann M. H., Bolser D. C. (2006). Diagnosis and management of cough executive summary: ACCP evidence-based clinical practice guidelines. *Chest*.

[83] Byamukama R., Barbara G., Namukobe J., Heydenreich M., Kiremire B. T. (2015). Bioactive compounds in the stem bark of *Albizia coriaria* (Welw. ex Oliver). *International Journal of Brain and Cognitive Sciences*.

[84] Wal P., Wal A., Sharma G., Rai A. (2011). Biological activities of lupeol. *Systematic Reviews in Pharmacy*.

[85] Vasconcelos J. F., Teixeira M. M., Barbosa-Filho J. M. (2008). The triterpenoid lupeol attenuates allergic airway inflammation in a murine model. *International Immunopharmacology*.

[86] Wu H., Xu F., Wang Y., Qian H., Wang X. (2017). Influence of general situation, glucose tolerance and insulin tolerance for lupenone in insulin resistance of type 2 diabetes rats. *Lishizhen Med Mater Med Res*.

[87] Nader M. A., Baraka H. N. (2012). Effect of betulinic acid on neutrophil recruitment and inflammatory mediator expression in lipopolysaccharide-induced lung inflammation in rats. *European Journal of Pharmaceutical Sciences*.

[88] Botha C., Penrith M.-L. (2008). Poisonous plants of veterinary and human importance in southern Africa. *Journal of Ethnopharmacology*.

[89] Randrianarivo H. R., Razafindrakoto A. R., Ratsimanohatra H. C. (2014). Toxic effects of seed methanolic extracts of endemic Albizia species (Fabaceae) from Madagascar on animals. *Journal of Life Sciences*.

[90] Kigondu E. V., Rukunga G. M., Keriko J. M. (2009). Anti-parasitic activity and cytotoxicity of selected medicinal plants from Kenya. *Journal of Ethnopharmacology*.

[91] Schultz F., Anywar G., Wack B., Quave C. L., Garbe L.-A. (2020). Ethnobotanical study of selected medicinal plants traditionally used in the rural Greater Mpigi region of Uganda. *Journal of Ethnopharmacology*.

[92] Shah K., Patel M., Patel R., Parmar P. (2010). Mangifera indica (mango). *Pharmacognosy Reviews*.

[93] Amin A. (2019). AL dulaimi and marwa AA AL rawi, antiviral activity of Mangifera extract on influenza virus cultivated in different cell cultures. *Journal of Pure and Applied Microbiology*.

[94] Zhu X., Song J., Huang Z., Wu Y., Yu M. (1993). Antiviral activity of mangiferin against herpes simplex virus type 2 in vitro. *Zhongguo yao li xue bao= Acta Pharmacologica Sinica*.

[95] Mushore J., Matuvhunye M. (2013). Antibacterial properties of *Mangifera indica* on *Staphylococcus aureus*. *African Journal of Clinical and Experimental Microbiology*.

[96] Alok P., Keerthana V., Kumar J. C., Ratan K., Chand A. D. (2013). Antibacterial property of two different varieties of Indian mango (*Mangifera indica*) kernel extracts at various concentrations against some human pathogenic bacterial strains. *Research Journal of Biological Sciences*.

[97] Doughari J., Manzara S. (2008). In vitro antibacterial activity of crude leaf extracts of *Mangifera indica* Linn. *African Journal of Microbiology Research*.

[98] Reddeman R. A., Glávits R., Endres J. R. (2019). A toxicological evaluation of mango leaf extract (*Mangifera indica*) containing 60% mangiferin. *Journal of Toxicology*.

[99] Garrido G., Rodeiro I., Hernández I. (2009). In vivo acute toxicological studies of an antioxidant extract from *Mangifera indica* L.(Vimang). *Drug and Chemical Toxicology*.

[100] Tiwari V., Darmani N. A., Yue B. Y., Shukla D. (2010). In vitro antiviral activity of neem (*Azardirachta indica* L.) bark extract against herpes simplex virus type‐1 infection. *Phytotherapy Research*.

[101] Badam L, Joshi S. P, Bedekar S. S (1999). ‘In vitro’ antiviral activity of neem (*Azadirachta indica*. A. Juss) leaf extract against group B coxsackieviruses. *Journal of Communicable Diseases*.

[102] Yerima M., Jodi S., Oyinbo K. (2012). Effect of neem extracts (*Azadirachta indica*) on bacteria isolated from adult mouth. *Nigerian Journal of Basic and Applied Sciences*.

[103] Kumari P. D., Shenoy S. M., Khijmatgar S., Chowdhury A., Lynch E., Chowdhury C. R. (2019). Antibacterial activity of new atraumatic restorative treatment materials incorporated with *Azadirachta indica* (Neem) against *Streptococcus mutans*. *Journal of oral biology and craniofacial research*.

[104] Blum F. C., Singh J., Merrell D. S. (2019). In vitro activity of neem (*Azadirachta indica*) oil extract against *Helicobacter pylori*. *Journal of Ethnopharmacology*.

[105] Maan P., Yadav K. S., Yadav N. P. (2017). Wound healing activity of *Azadirachta indica* A. juss stem bark in mice. *Pharmacognosy Magazine*.

[106] Mishra A., Dave N. (2013). Neem oil poisoning: case report of an adult with toxic encephalopathy. *Indian Journal of Critical Care Medicine: Peer-Reviewed, Official Publication of Indian Society of Critical Care Medicine*.

[107] Bakr S. A. (2013). Evaluation of acute toxicity of water extract of Azadirachta indica leaves and seeds in rats. *Pakistan Journal of Biological Sciences: PJBS*.

[108] Asikainen S., Alaluusua S. (1993). Bacteriology of dental infections. *European Heart Journal*.

[109] Chaieb K., Hajlaoui H., Zmantar T. (2007). The chemical composition and biological activity of clove essential oil, Eugenia caryophyllata (*Syzigium aromaticum* L. Myrtaceae): a short review. *Phytotherapy Research: An International Journal Devoted to Pharmacological and Toxicological Evaluation of Natural Product Derivatives*.

[110] Park S.-H., Sim Y.-B., Lee J.-K. (2011). The analgesic effects and mechanisms of orally administered eugenol. *Archives of Pharmacal Research*.

[111] Besra M., Kumar V. (2018). In vitro investigation of antimicrobial activities of ethnomedicinal plants against dental caries pathogens. *3 Biotech*.

[112] Nzeako B., Al-Kharousi Z. S., Al-Mahrooqui Z. (2006). Antimicrobial activities of clove and thyme extracts. *Sultan Qaboos University Medical Journal*.

[113] Prashar A., Locke I. C., Evans C. S. (2006). Cytotoxicity of clove (*Syzygium aromaticum*) oil and its major components to human skin cells. *Cell Proliferation*.

[114] Sarrami N., Pemberton M., Thornhill M., Theaker E. (2002). Adverse reactions associated with the use of eugenol in dentistry. *British Dental Journal*.

[115] Vijayasteltar L., Nair G. G., Maliakel B., Kuttan R., Krishnakumar I. (2016). Safety assessment of a standardized polyphenolic extract of clove buds: subchronic toxicity and mutagenicity studies. *Toxicology reports*.

[116] Roberts D. B., Travis E. L. (1995). Acemannan-containing wound dressing gel reduces radiation-induced skin reactions in C3H mice. *International Journal of Radiation Oncology, Biology, Physics*.

[117] Byeon S. W., Pelley R. P., Ullrich S. E., Waller T. A., Bucana C. D., Strickland F. M. (1998). Aloe barbadensis extracts reduce the production of interleukin-10 after exposure to ultraviolet radiation. *Journal of Investigative Dermatology*.

[118] Peng S., Norman J., Curtin G., Corrier D., McDaniel H., Busbee D. (1991). Decreased mortality of Norman murine sarcoma in mice treated with the immunomodulator, Acemannan. *Molecular Biotherapy*.

[119] Vahedi G., Taghavi M., Maleki A. K., Habibian R. (2011). The effect of *Aloe vera* extract on humoral and cellular immune response in rabbit. *African Journal of Biotechnology*.

[120] Ojiezeh T. I., Eghafona N. (2015). Humoral responses of broiler chickens challenged with NDV following supplemental treatment with extracts of *Aloe vera*, *Alma millsoni*, *Ganoderma lucidum* and *Archachatina marginata*. *Central-European Journal of Immunology*.

[121] Sydiskis R., Owen D., Lohr J., Rosler K., Blomster R. (1991). Inactivation of enveloped viruses by anthraquinones extracted from plants. *Antimicrobial Agents and Chemotherapy*.

[122] Lombardi N., Bettiol A., Crescioli G. (2020). Association between anthraquinone laxatives and colorectal cancer: protocol for a systematic review and meta-analysis. *Systematic Reviews*.

[123] Bolkent S., Akev N., Ozsoy N. (2004). Effect of Aloe vera (L.) Burm. fil. leaf gel and pulp extracts on kidney in type-II diabetic rat models.

[124] Lee A., Chui P. T., Aun C. S., Gin T., Lau A. S. (2004). Possible interaction between sevoflurane and *Aloe vera*. *The Annals of Pharmacotherapy*.

[125] Luyckx V. A., Ballantine R., Claeys M. (2002). Herbal remedy-associated acute renal failure secondary to Cape aloes. *American Journal of Kidney Diseases*.

[126] Wang W., Cuyckens F., Van den Heuvel H. (2003). Structural characterization of chromone C‐glucosides in a toxic herbal remedy. *Rapid Communications in Mass Spectrometry*.

[127] Rabe C., Musch A., Schirmacher P., Kruis W., Hoffmann R. (2005). Acute hepatitis induced by an *Aloe vera* preparation: a case report. *World Journal of Gastroenterology: WJG*.

[128] Evangelos C., Spyros K., Spyros D. (2005). Henoch-Schonlein purpura associated with Aloe vera administration. *European Journal of Internal Medicine*.

[129] Ernst E. (2000). Adverse effects of herbal drugs in dermatology. *British Journal of Dermatology*.

[130] Zhou Y., Feng Y., Wang H., Yang H. (2003). 90-day subchronic toxicity study of aloe whole-leaf powder. *Wei sheng yan jiu= Journal of hygiene research*.

[131] López Z., Femenia A., Núñez-Jinez G. (2019). In vitro immunomodulatory effect of food supplement from *Aloe vera*. *Evidence-based Complementary and Alternative Medicine*.

[132] Olila D., Opuda-Asibo J. (2001). Antibacterial and antifungal activities of extracts of *Zanthoxylum chalybeum* and *Warburgia ugandensis*, Ugandan medicinal plants. *African Health Sciences*.

[133] Merawie Y., Sahile S., Moges F., Husen A. (2013). Antimicrobial activity of crude and semi-purified fractions of *Warburgia ugandensis* against some pathogens. *Journal of Coastal Life Medicine*.

[134] Leonard C. M., Viljoen A. M. (2015). Warburgia: a comprehensive review of the botany, traditional uses and phytochemistry. *Journal of Ethnopharmacology*.

[135] Maroyi A. (2014). The genus Warburgia: a review of its traditional uses and pharmacology. *Pharmaceutical Biology*.

[136] Rabe T., Van Staden J. (2000). Isolation of an antibacterial sesquiterpenoid from Warburgia salutaris. *Journal of Ethnopharmacology*.

[137] Schultz F., Osuji O. F., Wack B., Anywar G., Garbe L.-A. (2021). Antiinflammatory medicinal plants from the Ugandan greater mpigi region act as potent inhibitors in the COX-2/PGH2 pathway. *Plants*.

[138] Karani L., Tolo F., Karanja S., Khayeka C. (2013). Safety of *Prunus africana* and *Warburgia ugandensis* in asthma treatment. *South African Journal of Botany*.

[139] Mwitari P. G., Ayeka P. A., Ondicho J., Matu E. N., Bii C. C. (2013). Antimicrobial activity and probable mechanisms of action of medicinal plants of Kenya: *Withania somnifera*, *Warbugia ugandensis*, *Prunus africana* and *Plectrunthus barbatus*. *PLoS One*.

[140] Anywar G., Kakudidi E., Byamukama R. t. (2021). A review of the toxicity and phytochemistry of medicinal plant species used by herbalists in treating people living with HIV/AIDS in Uganda. *Frontiers in Pharmacology*.

[141] Mao Q., Xu X., Cao S. (2019). Bioactive compounds and bioactivities of ginger (zingiber officinale roscoe). *Foods*.

[142] Zhang M., Viennois E., Prasad M. (2016). Edible ginger-derived nanoparticles: a novel therapeutic approach for the prevention and treatment of inflammatory bowel disease and colitis-associated cancer. *Biomaterials*.

[143] Zehsaz F., Farhangi N., Mirheidari L. (2014). The effect of *Zingiber officinale* R. rhizomes (ginger) on plasma pro-inflammatory cytokine levels in well-trained male endurance runners. *Central-European Journal of Immunology*.

[144] Suekawa M., Ishige A., Yuasa K., Sudo K., Aburada M., Hosoya E. (1984). Pharmacological studies on ginger. I. Pharmacological actions of pungent constituents,(6)-gingerol and (6)-shogaol. *Journal of Pharmacobio-Dynamics*.

[145] Akoachere J. T., Ndip R., Chenwi E., Ndip L., Njock T., Anong D. (2002). Antibacterial effects of Zingiber Officinale and Garcinia Kola on respiratory tract pathogens. *East African Medical Journal*.

[146] Chakotiya A. S., Tanwar A., Narula A., Sharma R. K. (2017). *Zingiber officinale*: its antibacterial activity on *Pseudomonas aeruginosa* and mode of action evaluated by flow cytometry. *Microbial Pathogenesis*.

[147] Saffari F., Ardakani M. D., Zandi H., Heidarzadeh H., Moshafi M. H. (2015). The effects of chlorhexidine and persica mouthwashes on colonization of *Streptococcus mutans* on fixed orthodontics O-rings. *Journal of Dentistry*.

[148] San Chang J., Wang K. C., Yeh C. F., Shieh D. E., Chiang L. C. (2013). Fresh ginger (*Zingiber officinale*) has anti-viral activity against human respiratory syncytial virus in human respiratory tract cell lines. *Journal of Ethnopharmacology*.

[149] Abd El-Wahab A., El-Adawi H., El-Demellawy M. (2009). In vitro study of the antiviral activity of *Zingiber officinale*. *Planta Medica*.

[150] Kaushik S., Jangra G., Kundu V., Yadav J. P., Kaushik S. (2020). Anti-viral activity of *Zingiber officinale* (Ginger) ingredients against the Chikungunya virus. *Virusdisease*.

[151] Plengsuriyakarn T., Viyanant V., Eursitthichai V. (2012). Cytotoxicity, toxicity, and anticancer activity of *Zingiber officinale* Roscoe against cholangiocarcinoma. *Asian Pacific Journal of Cancer Prevention*.

[152] Rong X., Peng G., Suzuki T., Yang Q., Yamahara J., Li Y. (2009). A 35-day gavage safety assessment of ginger in rats. *Regulatory Toxicology and Pharmacology*.

[153] Shalaby M., Hamowieh A. (2010). Safety and efficacy of *Zingiber officinale* roots on fertility of male diabetic rats. *Food and Chemical Toxicology*.

[154] Weidner M. S., Sigwart K. (2000). Investigation of the teratogenic potential of a *Zingiber officinale* extract in the rat. *Reproductive Toxicology*.

[155] Cellini L., Di Campli E., Masulli M., Di Bartolomeo S., Allocati N. (1996). Inhibition of *Helicobacter pylori* by garlic extract (*Allium sativum*). *FEMS Immunology and Medical Microbiology*.

[156] Sivam G., Lampe J., Ulness B. (1996). *Helicobacter pylori* in vitro susceptibility to garlic (*Allium sativum*). *FEMS Immunology and Medical Microbiology*.

[157] Mohammed A., Mohammed A., Prasad V. S. (2009). Antiulcer activity of *Allium sativum* bulb juice in rats. *Saudi Pharmaceutical Journal*.

[158] Mubarak M. S., Hadda T. B., ElSawy N. A., Header E. A., Mabkhot Y. N. (2014). Effect of garlic and cabbage on healing of gastric ulcer in experimental rats. *Medicinal Chemistry Research*.

[159] Savitri N. H., Indiastuti D. N., Wahyunitasari M. R. (2019). Inhibitory activity of *Allium sativum* L. extract against Streptococcus pyogenes and *Pseudomonas aeruginosa*. *Journal of Vocational Health Studies*.

[160] Chen C.-H., Chou T.-W., Cheng L.-H., Ho C.-W. (2011). In vitro anti-adenoviral activity of five Allium plants. *Journal of the Taiwan Institute of Chemical Engineers*.

[161] Mehrbod P., Aini I., Amini E. (2013). Assessment of direct immunofluorescence assay in detection of antiviral effect of garlic extract on influenza virus. *African Journal of Microbiology Research*.

[162] Mehrbod P., Amini E., Tavassoti-Kheiri M. (2009). Antiviral activity of garlic extract on influenza virus. *Iranian Journal of Virology*.

[163] Weber N. D., Andersen D. O., North J. A., Murray B. K., Lawson L. D., Hughes B. G. (1992). In vitro virucidal effects of *Allium sativum* (garlic) extract and compounds. *Planta Medica*.

[164] Rouf R., Uddin S. J., Sarker D. K. (2020). Anti-viral potential of garlic (*Allium sativum*) and it’s organosulfur compounds: a systematic update of pre-clinical and clinical data. *Trends in Food Science & Technology*.

[165] Stevinson C., Pittler M. H., Ernst E. (2000). Garlic for treating hypercholesterolemia: a meta-analysis of randomized clinical trials. *Annals of Internal Medicine*.

[166] Asero R., Mistrello G., Roncarolo D., Antoniotti P. L., Falagiani P. (1998). A case of garlic allergy. *The Journal of Allergy and Clinical Immunology*.

[167] Perez‐Pimiento A., Santaolalla M., De Paz S., Fernández‐parra B., Domínguez‐lázaro A., Moneo I. (1999). Anaphylactic reaction to young garlic. *Allergy*.

[168] Pires G., Pargana E., Loureiro V., Almeida M., Pinto J. (2002). Allergy to garlic. *Allergy*.

[169] Burden A., Wilkinson S., Beck M., Chalmers R. (1994). Garlic-induced systemic contact dermatitis. *Contact Dermatitis*.

[170] Izzo A. A. (2005). Herb–drug interactions: an overview of the clinical evidence. *Fundamental & Clinical Pharmacology*.

[171] Brochot A., Guilbot A., Haddioui L., Roques C. (2017). Antibacterial, antifungal, and antiviral effects of three essential oil blends. *Microbiologyopen*.

[172] Goldbeck J. C., do Nascimento J. E., Jacob R. G., Fiorentini Â. M., da Silva W. P. (2014). Bioactivity of essential oils from *Eucalyptus globulus* and *Eucalyptus urograndis* against planktonic cells and biofilms of *Streptococcus mutans*. *Industrial Crops and Products*.

[173] Ambrosio C. M. S., de Alencar S. M., Moreno A. M., Da Gloria E. M. (2018). Evaluation of the selective antibacterial activity of *Eucalyptus globulus* and *Pimenta pseudocaryophyllus* essential oils individually and in combination on *Enterococcus faecalis* and *Lactobacillus rhamnosus*. *Canadian Journal of Microbiology*.

[174] Khozeimeh F., Golestannejad Z., Seifi S., Pourarian A., Gavanji S., Farhad F. (2015). Chemical composition and in vitro antibacterial activities of *Cuminum cyminum* L. and *Eucalyptus globulus* essential oil extracts against three lactobacillus strains. *Biological Research*.

[175] Silva J., Abebe W., Sousa S., Duarte V., Machado M., Matos F. (2003). Analgesic and anti-inflammatory effects of essential oils of Eucalyptus. *Journal of Ethnopharmacology*.

[176] Hu Z., Feng R., Xiang F. (2014). Acute and subchronic toxicity as well as evaluation of safety pharmacology of eucalyptus oil-water emulsions. *International Journal of Clinical and Experimental Medicine*.

[177] Mengiste B., Zenebe T., Dires K. (2020). Safety evaluation of *Eucalyptus globulus* essential oils through acute and sub-acute toxicity and skin irritation in mice and rats. *Current Chemical Biology*.

